# Phagocytosis, Degranulation and Extracellular Traps Release by Neutrophils—The Current Knowledge, Pharmacological Modulation and Future Prospects

**DOI:** 10.3389/fphar.2021.666732

**Published:** 2021-05-04

**Authors:** Barbara Gierlikowska, Albert Stachura, Wojciech Gierlikowski, Urszula Demkow

**Affiliations:** ^1^ Department of Laboratory Diagnostics and Clinical Immunology of Developmental Age, Medical University of Warsaw, Warsaw, Poland; ^2^ Department of Methodology, Centre for Preclinical Research, Medical University of Warsaw, Warsaw, Poland; ^3^ Doctoral School, Medical University of Warsaw, Warsaw, Poland; ^4^ Department of Internal Medicine and Endocrinology, Medical University of Warsaw, Warsaw, Poland

**Keywords:** neutrophils, phagocytosis, degranulation, extracellular traps, inflammation

## Abstract

Neutrophils are crucial elements of innate immune system, which assure host defense via a range of effector functions, such as phagocytosis, degranulation, and NET formation. The latest literature clearly indicates that modulation of effector functions of neutrophils may affect the treatment efficacy. Pharmacological modulation may affect molecular mechanisms activating or suppressing phagocytosis, degranulation or NET formation. In this review, we describe the role of neutrophils in physiology and in the course of bacterial and viral infections, illustrating the versatility and plasticity of those cells. This review also focus on the action of plant extracts, plant-derived compounds and synthetic drugs on effector functions of neutrophils. These recent advances in the knowledge can help to devise novel therapeutic approaches via pharmacological modulation of the described processes.

## Neutrophils in Health and Disease

### Role in Health

**FIGURE 1:**
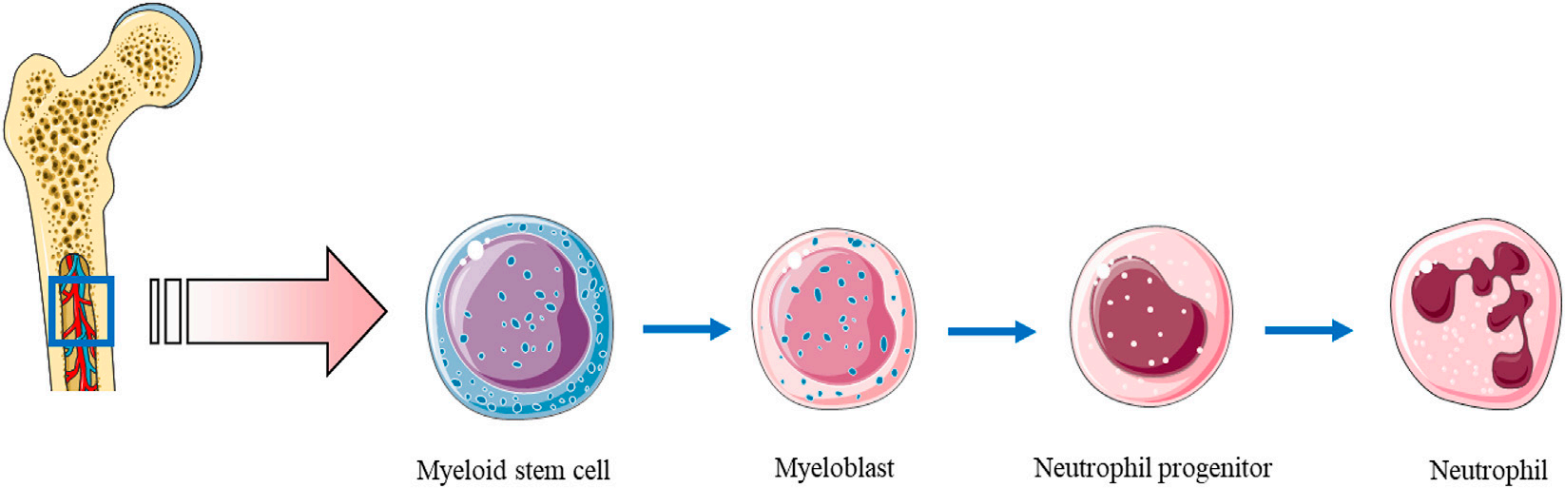
The granulocyte formation.

Neutrophils are produced in the venous sinuses of the bone marrow, where they derive from a common myeloid progenitor cells (Figure 1; Nerlov and Graf, 1998). Recently, a novel classification of bone marrow neutrophil-lineage cells emerged. Progenitor stem cells evaluate into a pool of preneutrophils, further differentiating into non-proliferative immature neutrophils and, eventually, mature neutrophils, equipped with numerous machineries to combat pathogens. Maturation of this lineage is orchestrated by transcription factors such as GFI1 and Pu.1 (Evrard et al., 2018). Neutrophils are continually generated in the bone marrow (daily production may reach up to 2 × 10^11^ cells), where also begin the maturation process (Borregaard, 2010). Granulocyte-colony stimulating factor (G-CSF) is the main agent responsible for their development, production and release (Lieschke et al., 1994). *In vivo* studies showed that neutrophils may circulate in human blood for ∼10 h (Athens et al., 1961a; McMillan and Scott, 1968) to 5 days (Pillay et al., 2010). Nonetheless, in the course of the development of inflammatory reaction, neutrophils become active, and their lifespan is extended. This is a result of interactions with various cytokines, (e.g. IL-1 beta, tumor necrosis factor (TNF), IFN-gamma), as well as bacterial products, such as lipopolysaccharide (LPS) (Colotta et al., 1992; Summers et al., 2010; Kim et al., 2011). Mature neutrophils (with segmented nuclei and fully formed granules) may also proliferate outside of the bone marrow—in the spleen after infection (Deniset et al., 2017) or when they come in contact with serum amyloid A (De Santo et al., 2010). Longer lifespan allows them to shape many processes: tissue healing, inflammation resolution and modulation of adaptive immune responses, but also may cause adverse effects, including tissue damage (Liew and Kubes, 2019). Neutrophils’ maturation and aging goes in parallel with natural drift in their phenotype and function in the absence of inflammation. These cells undergo daily modifications (Casanova-Acebes et al., 2013), such as: expression of adhesion molecules affecting tissue trafficking, (e.g. CD62L, CD11b, and CD49 days), expression of chemokines receptors, (e.g. CXCR2 and CXCR4), pathogen receptors (Toll-like and NOD-like receptors), activity of pathways related to cell activation (such as NF-kB and MAPK signaling), and cell death (as revised in (Adrover et al., 2016)). This process is influenced by the microbiome, as its depletion with broad-spectrum antibiotics reduces neutrophil number and aging, which can be reversed by LPS stimulation. Similar results were observed in germ-free and TLR2, TLR4, and Myd88 knock-out mice. Neutrophils not stimulated by microbiota are less responsive to pathogens, e.g., their NET formation is decreased (Zhang et al., 2015). The circadian cycle affects the function of neutrophils, possibly as a result of cyclic release of newly formed cells from the bone marrow (Ella et al., 2018). Such a regulation has an impact on the neutrophils’ functions in immunity and inflammation (He et al., 2018).

Over 50 years ago, study of Mauer et al. showed that granulocytes rapidly disappear from circulation when injected into healthy volunteers (Mauer et al., 1960), with further conclusion that total blood granulocyte pool is made up of two compartments, i.e., circulating granulocyte pool and marginal granulocyte pool. They are in an equilibrium with each other and it is possible to move cells from the latter to the former compartment by stimuli like epinephrine or physical exercise (Athens et al., 1961b). Further studies revealed that in absence of inflammation neutrophils can be found in other compartments, including bone marrow (where they inhibit hematopoietic niches and trigger the release of hematopoietic progenitor cells into the bloodstream), spleen (mainly in the marginal zone and red pulp; supporting B cell maturation and antibody production), liver, lung (where they prevent formation of melanoma metastases and affect lung transcriptome in diurnal pattern), intestine (where phagocytosis of incoming neutrophils by resident macrophages regulates cytokine production and participates in the remote regulation of bone marrow niches), white adipose tissue, skin, skeletal muscle, peripheral lymph nodes, intestine (as isolated patches in the intestine) kidneys, and heart. Only immune-privileged organs, i.e., ovaries, testes, and brain are free from neutrophils. Apoptotic neutrophils located in skin, muscle, and intestine are phagocyted by dendritic cells and macrophages, which inhibits IL-23 expression and thus controls granulopoiesis by regulating G-CSF production (Casanova-Acebes et al., 2013). How neutrophils persist in these organs is not fully elucidated yet. Intravascular transit time in the liver is around 2 min (Peters et al., 1985), in the spleen and bone marrow—10 min (Peters et al., 1985; Ussov et al., 1995). Recently, two pools of neutrophils were identified in the spleen. Mobile neutrophils were roaming around the red pulp, while the stationary ones remained in the perivascular area, both populations differ in terms of Ly6G expression. Mature, (i.e. Ly6G^high^) splenic neutrophils were necessary to eradicate *Staphylococci* during systemic infection. This process takes place mainly in the red pulp, whereas immature neutrophils, (i.e. Ly6G^int^), residing perivascular area, undergo emergency proliferation and mobilization (Deniset et al., 2017) Using intravital microscopy large quantities of neutrophils resident in the lung under normal conditions were visualized (Kreisel et al., 2010). Other study showed that neutrophils in this location are not simply “stuck” in the capillary bed, but rather are on the move–tethering, crawling or adhered to cell surfaces (Yipp et al., 2017). Lung is an organ with a high number of neutrophils in marginated pool, maintained due to expression of adhesion molecules on neutrophils themselves and on endothelial cells. After stimulation, such as infection, profile of adhesion molecules evolve quickly, allowing transmigration (Sibille and Marchandise, 1993). Moreover, the persistence time in this organ is way longer than the mean neutrophil intravascular transit time estimated for other organs (Peters et al., 1985; Lien et al., 1987; Ussov et al., 1995).

Granulocytes’ clearance from the circulation takes place mainly in the liver, spleen, and bone marrow (Shi et al., 2001; Hong et al., 2012). As they grow older, the expression of CXC-chemokine receptor 4 (CXCR4) increases, which probably leads them back to the bone marrow, where they are eliminated (Casanova-Acebes et al., 2013). The same receptor also negatively regulates release of the newly formed neutrophils from the bone marrow (Eash et al., 2009; Summers et al., 2010). CXCR4 is not the only factor responsible for the destruction of neutrophils as cells that lack this receptor have the same half-life as the wild type neutrophils (Eash et al., 2009). Granulocytes may also die in the blood vessels and are later removed by the Kupffer cells (liver-resident macrophages) (Shi et al., 2001). The neutrophils clearance by Kupffer or dendritic cells is regulated, in part, by the IL-23, IL-17 and granulocyte colony-stimulating factor (G-CSF) cytokine axis. These mediators stimulate granulocyte production in the bone marrow as well (Stark et al., 2005). There are still significant gaps in the knowledge about the neutrophil clearance by other cells. Neutrophils die primarily by an intrinsic mechanism of apoptosis, regulated by Bcl-2 protein family. MCL-1 is the key anti-apoptotic agent, antagonizing the pro-apoptotic effects of remaining Bcl-2 family members. Severe neutropenia without affecting other cells circulating in the blood was observed in MCL-1 knock-out mice (Csepregi et al., 2018). During the resolution of inflammation, macrophages dispose apoptotic neutrophils by phagocytosis, releasing lipid mediators. These molecules play a role in re-establishing homeostasis and their absence likely leads to chronic inflammation (Buckley et al., 2014). Some neutrophils sacrifice themselves, releasing neutrophil extracellular traps (NETs) to fight pathogens (Brinkmann et al., 2004; Stark et al., 2005). Neutrophils are also capable of vital netosis–i.e., releasing NETs by a living cell (Yipp and Kubes, 2013).

Significant heterogeneity and functional versatility are observed among neutrophils (Scapini et al., 2016; Silvestre-Roig et al., 2016; Deniset and Kubes, 2018). Both, in steady state and in the course of inflammation, these cells undertake various roles, not always beneficial. It is still unclear whether this variety results from different programs of neutrophils’ maturation and activation or is somehow influenced by external factors. In patients with autoimmune disorders, low density neutrophils/granulocytes were more frequent, though their functions are not precisely defined (Hacbarth and Kajdacsy-Balla, 1986; Scapini et al., 2016). Polymorphonuclear myeloid-derived suppressor cells (PMN-MDSCs) are responsible for the failure of many cancer therapies and poor clinical outcomes. They derive from pathologically activated neutrophils, however, the exact mechanism is of their action is unclear (Gabrilovich, 2017). Two distinct fractions of tumor-associated neutrophils are identified in cancer patients. N1—a pro-inflammatory and anti-tumor subset, induced by TGF-β blockade and N2–a protumor group, increasing in number, following stimulation by TGF-β (Fridlender et al., 2009). The concept of neutrophils’ heterogeneity cannot be universally summarized. We still see the vast gaps in the knowledge that preclude a succinct explanation for this phenomenon. Nevertheless, this heterogeneity may have a significant impact on choosing potential targets for future therapeutic agents (Nemeth et al., 2020).

To exert their effects, neutrophils must first reach the target tissue. The recruitment of granulocytes into the inflamed site involves tethering, rolling, adhesion, crawling and transmigration (Kolaczkowska and Kubes, 2013). The entire process is initiated via activated endothelium that exposes adhesion molecules, that enable leukocytes recruitment. Activation occurs via stimulation by mediators such as histamine, cysteinyl-leukotrienes and cytokines, usually released by resident leukocytes, when the pathogens are present (Ley et al., 2007). Endothelial cells may also be activated directly. If pattern-recognition receptor (PRR) connects with its ligand, the number of adhesion molecules increases on the endothelium surface. P-selectin and E-selectin are responsible for further neutrophil recruitment steps (Petri et al., 2008). They bind to their ligands, including P-selectin glycoprotein ligand 1 (PSGL1), capturing free-flowing neutrophils to the endothelium surface and promote subsequent granulocytes’ rolling along the vessel. Adhesion, crawling and transmigration depend, to a large extent, on integrin interactions with cell adhesion molecules (CAMs) (Phillipson et al., 2006). Luminal surface of endothelium exposes chemokines, which activate rolling neutrophils, thus inducing conformational changes and completing the extravasation process. Neutrophils are capable of returning to the bloodstream via a process called reverse transendothelial cell migration (rTEM). Neutrophils that underwent rTEM are characterized by high CD54 and low CXCR1 expression and are identified more frequently in case of systemic inflammation than in healthy donors (Buckley et al., 2006). Other forms of neutrophils’ motility are: 1) reverse luminal crawling—moving along blood vessel against blood flow; 2) reverse abluminal crawling—occurs while seeking essential directional cues to fully breach venular walls and 3) reverse interstitial migration–a movement directed away from inflammation site within interstitium, which may result in remote organ damage, as documented in ischemic injury (Nourshargh et al., 2016).

### Role in Disease

Neutrophils play key roles in many diseases, protecting against pathogens and regulating innate and adaptive immunity. On the other hand, when hyperactive or abnormally stimulated, they may lead to tissue damage and exacerbate existing pathology.

During sepsis, neutrophils show enhanced responsiveness to chemokines, resulting in their accumulation at the infection site (Angus et al., 2001; Martin et al., 2003; Sônego et al., 2016). Some hypotheses suggest that sepsis is associated with an early overwhelming innate immune response, characterized by dysregulation of the overproduction of cytokines (TNF-α, IL-1β, IL-6, IL-8) (Benjamim et al., 2004; Faix, 2013; Chousterman et al., 2017). Concentrations of circulating pro-inflammatory cytokines are low or undetectable in healthy individuals but their production is stimulated during invasion by pathogenic microorganisms. In human and experimental animal models of sepsis, cytokines are released in a sequential manner resulting in a “cytokine cascade” (Steinhauser et al., 1999). It is initiated when a stimulus, such as Gram-negative bacterial endotoxin, (e.g. lipopolysaccharides released by *E. coli*), induces production of the “early inflammatory cytokines,” like TNF-α and IL-1β. TNF-α was shown to be a central factor of immune regulation and mediator of the pathophysiological changes associated with bacteremia and sepsis syndrome (Benjamim et al., 2004). Overproduction of TNF-α correlates with enhanced properties of phagocytes. In contrast, IL-1β serum levels are only slightly increased during sepsis. The release of “early inflammatory cytokines,” intensifies the production of the “late inflammatory cytokines”—IL-6 and IL-8. IL-6 was recognized as a marker of sepsis with high specificity (Kuster et al., 1998). The increased plasma IL-8 concentration in adult sepsis-occurring patients may correlate with mortality (Marty et al., 1994). Although various pro-inflammatory cytokines contribute to the inflammatory cascade, other cytokines also display anti-inflammatory properties, serving to counterbalance a potentially inadequate pro-inflammatory state. In sepsis, interleukin 10 has been shown to act as the primary endogenous modulator of inflammatory response.

The dysregulation of “cytokine balance” influences of the immune cells functions including phagocytosis and NETosis. The question is: how may cytokine profile affect those processes? Where is the balance between “too much” and “not enough?” A similar question was asked by Garner and colleagues (Garner et al., 1996), who showed that the increasing concentration of TNF-α and IL-1β may suddenly upregulate Fc receptor (FcγR)-mediated phagocytosis by human polymorphonuclear neutrophils (PMN). Nevertheless, the mechanisms of this enhanced phagocytosis remain unknown. This same issue was investigated by Erwig and colleagues. They exposed macrophages on single cytokine and cytokine mixture (IFN-γ, TNF-α, TGF-β, IL-4, IL-6, and IL-10). After 48 h their function was evaluated for nitric oxide (NO) generation, uptake of apoptotic neutrophils, and β-glucuronidase expression. The phagocytic properties of leukocytes were augmented by TNF-α (40 vs 29% controls) and decreased by IFN-γ, IL-10, and IL-4 (Erwig et al., 1998).

Similarly to phagocytosis, NET formation (Galluzzi et al., 2018) may be effective anti-microbial mechanism in the course of sepsis, however with time it may contribute to tissue and endothelial damage, finally leading to disseminated intravascular coagulation (DIC), with high mortality rate. Interestingly, both SOFA (Sequential Organ Failure Assessment) score and acute kidney injury (AKI) correlates with cell-free DNA, measured in septic patient serum (Klopf et al., 2021).

Some studies highlight neutrophils contribution to antiviral immunity (Saitoh et al., 2012; Jenne et al., 2013). In the course of COVID-19, neutrophils-to-lymphocytes ratio was established as an independent prognostic factor (Wang et al., 2020). Recent study showed that increased amounts of reactive oxygen species (ROS) and the release of NETs are associated with intensive thrombi formation–one of the most prevalent and serious COVID-19 complications (Arcanjo et al., 2020). In COVID-19 patients, cell-free DNA, MPO-DNA complexes, and citrullinated histone H3 have been assessed and appeared to be elevated. Interestingly, cell-free DNA and MPO-DNA complexes were significantly higher in the blood from mechanically ventilated vs. non-ventilated patients. It was suggested that vascular damage, resulting in acute respiratory distress syndrome (ARDS) and multiorgan dysfunction, may be due to NET formation (Klopf et al., 2021). The abovementioned findings may link cytokine storm (as discussed in (Ragab et al., 2020)), microangiopathic occlusions and ARDS, and justifies use of steroids, (i.e. dexamethasone, as shown in RECOVERY trial (Horby et al., 2021)) and tocilizumab (Zhang et al., 2020b) in COVID-19 treatment.

In the respiratory system, neutrophils act like a double-edged sword. On the one hand, they defend organism against pathogens, but on the other they cause extensive tissue damage in e.g., acute lung injury, chronic obstructive pulmonary disease, or neutrophilic asthma (Grommes and Soehnlein, 2011; Meijer et al., 2013; Krishnamoorthy et al., 2018). Neutrophils also contribute to formation and destabilization of atherosclerotic plaques (Soehnlein, 2012). The accumulation of these cells is associated with a greater risk of plaque rupture (Ionita et al., 2010). Moreover, neutrophils accumulated at the plaque site may release NETs, which leads to smooth muscle cells damage, prompting destabilization (Silvestre-Roig et al., 2019).

In ischemic diseases, such as myocardial infarction or stroke, inflammatory processes cause severe tissue damage, especially after reperfusion (Eltzschig and Eckle, 2011). In myocardial infarction model, Ly6C^high^ monocytes and neutrophils contributed comparably to proteolysis, which may result in left ventricle rupture (Anzai et al., 2017). As mentioned above, neutrophils may undergo rTEM, stimulated by LTB_4_, from area of ischemia-reperfusion injury and contribute to distant organ damage (Colom et al., 2015).

The role of NET formation in autoimmune disease was reviewed by Mitsios et al. (Mitsios et al., 2016) and Lee et al. (Lee et al., 2017). Some authors suggest that the anti-DNA immunization in systemic lupus erythematosus may be initiated by NETs. Released nucleic acid activates the dendritic cells, which in turn prompt antibodies production by B cells, causing systemic autoimmunity (Garcia-Romo et al., 2011; Lande et al., 2011). Consistently, citrullinated histones, present in NETs were suggested as the trigger activating anti-citrullinated peptide autoantibodies (ACPAs) production, prevalent in rheumatoid arthritis (Khandpur et al., 2013). Release of proteinase 3 (PR3) and myeloperoxidase (MPO) may initiate antineutrophil cytoplasmic antibody-associated vasculitis (Kessenbrock et al., 2009).

Cancer biology is influences by neutrophils’ behavior. As mentioned before, neutrophils may exert pro-inflammatory or pro-tumoral effects (Fridlender et al., 2009). This phenotypic switch and overall plasticity may explain the vast spectrum of neutrophil activities, both harmful and beneficial to the host (Sagiv et al., 2015). Some of the undesirable effects include suppression of T cell-mediated antitumor immunity (Coffelt et al., 2015; Steele et al., 2016) or elastase-mediated degradation of IRS1 (Houghton et al., 2010), enhancing cancer cell proliferation. On the other hand, some studies reported that neutrophils prevent metastasis formation (Granot et al., 2011) or inhibit early cancer growth (Blaisdell et al., 2015). In solid tumors, increased neutrophils number in tumor microenvironment is a negative outcome predictor (Templeton et al., 2014; Gentles et al., 2015).

## TO Phagocytose, to Degranulate or to Make Extracellular Traps?

**FIGURE 2:**
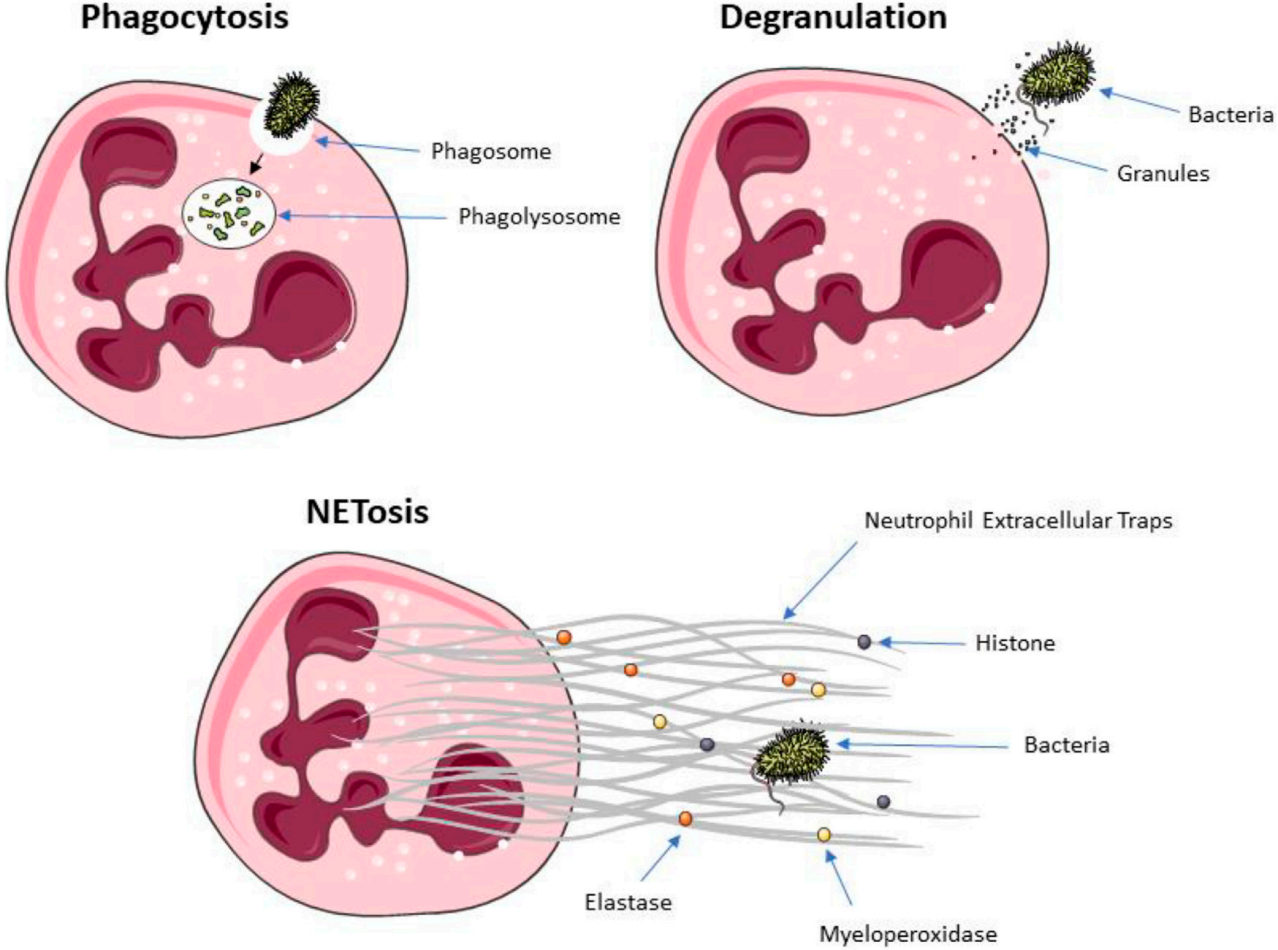
The killing mechanisms of neutrophils: phagocytosis, degranulation, and extracellular traps release.

Neutrophils are crucial elements of innate immune system, which assure host defense via a range of effector functions, such as phagocytosis, degranulation, and NET formation (Figure 2; Kolaczkowska and Kubes, 2013).

Phagocytosis is a fundamental mechanism for the effective elimination of disease-causing agents (Schumann, 2016). It enables clearance of microbes, but also dead cells and tissue debris (Jaumouillé and Waterman, 2020). Thus, phagocytosis is a fundamental component responsible for tissue homeostasis and the innate immune response (Jaumouillé and Waterman, 2020). This process is initialized by internalization of targeted organism/particle. Two different mechanisms have been identified for the internalization of diverse particulate material: 1) the trigger mechanism where discreet signaling initiates formation of plasma membrane protrusions, shaped by actin, that surround nearby material, and 2) the zipper mechanism where cell surface receptors sequentially bind to ligands on the target particle, which leads to a complete wrapping of the particle by the plasma membrane (Nordenfelt and Tapper, 2011). Only a small number of pathogens, (e.g., *Salmonella* or *Shigella*) is able to initiate the process, whereas the zipper mechanism, involving a wide spectrum of phagocytic receptors, allows to successfully bind many species of pathogen/particles (Jaumouillé and Waterman, 2020). The essential phagocytic receptors are Fcγ class receptors, which recognize IgG, the complement receptor 3 (αMβ2 integrin). While TLRs and NOD receptors are not classified as phagocytic receptors, their activation may increase phagocytosis (Nordenfelt and Tapper, 2011). Receptor activation initiates signaling cascades that re-model lipids in the cell membrane and leads to rearrangement of actin cytoskeleton in order to extend the cell membrane around the particle. After reconstructing the plasma membrane of the phagocyte, bacteria are ultimately internalized in phagosome (Fairn and Grinstein, 2012). The dynamics of phagosome maturation is highly dependent on the several regulators like Rab proteins synthesis. Among known Rab proteins, Rab5 and Rab7 are directly involved in regulation of phagosome functions (Mottola, 2014).

Unlike macrophages, maturation of neutrophilic phagosomes is dependent on fusion with preformed granules (Nordenfelt and Tapper, 2011; Gierlikowska et al., 2020b). Membrane proteins and granule contents are directed to distinct locations by precise trafficking and fusion/fission processes (Nordenfelt and Tapper, 2011). Activation of granules is assisted by recruitment of NADPH oxidase (Johansson et al., 1995). It is suggested that an early alkalization of neutrophil phagosomes leads to the oxidative burst in the phagosomes (Segal et al., 1981).

Besides ROS production, neutrophils eliminate pathogens via production of microbicidal molecules following degranulation (Mortaz et al., 2018).

Degranulation is important for pathogen killing, but also modulates the immune response during infectious or non-infectious diseases (Mortaz et al., 2018). Different granule types are released sequentially (Kolaczkowska and Kubes, 2013). At the beginning, tertiary granules are released, (e.g. cathepsin, gelatinase, known as metalloprotease 9), then secondary granules, (e.g. lactoferrin, alkaline phosphatase, lysozyme, cathelicidin, NADPH oxidase and collagenase). At the end, primary granules are secreted containing the most pro-inflammatory and antimicrobial proteins, such as elastase, MPO, cathepsin G, defensins (HNP-1, HNP-2 and HNP-3 peptides) and bacterial permeability increasing protein (BPI) (Faurschou and Borregaard, 2003). Normal neutrophil degranulation involves the release of primary and secondary granules mainly into the phagosome, which prevents host tissue damage (Sengeløv et al., 1995). Bacteria are able to disrupt, dysregulate, or induce excessive neutrophil degranulation, in order to diminish the protective effects of neutrophil degranulation in a way that ultimately benefits the pathogen and extend the disease course, thus pharmacological modulation may potentially bring promising results for recovering (Eichelberger and Goldman, 2020).

Neutrophils use a few strategies to counteract infections. Two of those strategies have been thoroughly studied and are described above: phagocytosis and degranulation. They can also utilize NADPH oxidase to form ROS which have antimicrobial potential (Mantovani et al., 2011). A new mechanism that combats infections—NET formation–has been described in 2004 (Brinkmann et al., 2004). Once the process is initiated, the chromatin in neutrophils’ DNA loosens up and it forms complexes with numerous granular and cytoplasmic proteins, which are subsequently released into the extracellular space. Depending on the origin of proteins, they could by classified as histones (H1, H2A, H2B, H3, and H4), azurophilic granules (MPO, elastase, defensins, cathepsin G), specific granules (lactoferrin, alkaline phosphatase, NADPH oxidase, lysozyme, collagenase), tertiary granules (gelatinase, cathepsin) and cytosol proteins (LL-37, proteinase 3, neutrophil serine protease 4 and tryptase) (Brinkmann et al., 2004). The biological function of extracellular traps is to sequester a specific space, enabling accumulation of high concentration of antimicrobial agents, preferably at the site of infection. Thus extracellular traps immobilize and kill microorganisms, which prevent them from dissemination (Manda et al., 2014).

Modulations of killing mechanisms (like phagocytosis, degranulation, or NET formation) could be of interest, as it may potentially boost pathogen killing or protect host from own tissue damage.

## Pharmacological Modulation of Phagocytosis via Medical Plants

Therapies targeted at innate immune response modulation via medicinal plants and metabolites isolated from them experience a renaissance in recent years (Ríos, 2010; Licciardi and Underwood, 2011; Grigore et al., 2020; Behl et al., 2021).

After performing a literature search using relevant MeSH terms and keywords, we identified 53 plant extracts, which were tested on neutrophil model and their influence on phagocytic properties were evaluated (Table 1). Selected plants belonged to diverse families: Anacardiaceae, Araceae, Araliaceae, Arecaceae, Apocynaceae, Asphodelaceae, Asteraceae, Boraginaceae, Capparaceae, Celastraceae, Convolvulaceae, Cucurbitaceae. Elaeocarpaceae, Euphorbiaceae, Fabaceae, Geraniaceae, Hypericaceae, Lamiaceae, Lythraceae, Malvaceae, Melastomataceae, Meliaceae, Menispermaceae, Moraceae, Moringaceae, Phyllanthaceae, Poaceae, Ranunculaceae, Rosaceae, Rubiaceae, Santalaceae, Sapindaceae, Saxifragaceae, Theaceae, Zingiberaceae, and Zosteraceae. Plant materials were collected in Europe, Asia, Southern and Northern America and Africa. The geographic location directly determines the phytochemical composition of plants and their pharmacological activity (Czerwińska and Melzig, 2018; Kiss et al., 2020). Thus, evaluation of the medical plants collected from different locations, may highlight future directions for searching other plants (with similar chemical composition), and then select them for compounds isolation (Gierlikowska et al., 2020a). We noticed that 33 of the selected plant extracts stimulated phagocytic activity of neutrophils, 18 decreased phagocytic activity and 2 extracts did not affect the process.

**TABLE 1:** List of selected plant material with documented impact on phagocytosis.

	Plant family	Species	Plant material	Cellular target and mechanism of action	Effect on phagocytosis	References
1	Anacardiaceae	Semecarpus anacardium L.f	Plant extract	Increase of reactive oxygen species production and lysosomal enzymes activity (acid phosphatase and cathepsin D)	Increased phagocytic activity	Ramprasath et al. (2006)
2	Araceae	Lemna minor L	Plant extract	n.d	Increased phagocytic activity	Hu et al. (1995)
3	Araliaceae	Panax ginseng C.A.Mey	Plant extract	n.d	Increased phagocytic activity	Hu et al. (1995)
4	Arecaceae	Areca catechu L	Plant extract	Suppression of CR1, CR3, CR4 and fcγ receptors expression, decrease of F-actin stability	Inhibition of phagocytic activity	Hung et al. (2006); Lee et al. (2014)
5	Apocynaceae	Leptadenia pyrotechnica (forssk.) decne	Plant extract	n.d	Increased phagocytic activity	Rasheed et al. (2016)
6	Asphodelaceae	Aloe vera (L.) Burm.f	Plant extract	Reduction the availability of intracellular free Ca^2+^ and inhibition of reactive oxygen species production	Increased phagocytic activity	Ou. (1991)
7	Asteraceae	Echinacea purpurea (L.) moench	Plant extract	Stimulation of C3b and fcγ receptors	Increased phagocytic activity and digestive capacity	Melchart et al. (1995); Yang et al. (2002); Isaykina et al. (2008)
8	Asteraceae	Echinacea pallida (nutt.) nutt	Plant extract	n.d	Increased phagocytic activity	Melchart et al. (1995)
9	Asteraceae	Anacyclus pyrethrum (L.) lag	Plant extract	n.d	Increased phagocytic activity	Sharma et al. (2010)
10	Asteraceae	Santolina chamaecyparissus L	Plant extract	n.d	Inhibited phagocytic activity	Boudoukha et al. (2016)
11	Asteraceae	Baccharis dracunculifolia DC.	Plant extract	n.d	Inhibited phagocytic activity	Figueiredo-Rinhel et al. (2017)
12	Asteraceae	Centaurea pumilio L	Plant extract	n.d	Increased phagocytic activity	Mostafa et al. (2019)
13	Boraginaceae	Echium amoenum fisch. and C.A.Mey	Plant extract	Increase of reactive oxygen species production	Increased phagocytic activity	Asadollahi et al. (2015)
14	Boraginaceae	Heliotropium sarmentosum (lam.) craven	Plant extract	Increase of MAC-1 cell surface expression and activation of AKT signaling pathway	Increased phagocytic activity	Chen et al. (2014)
15	Capparaceae	Capparis zeylanica L	Plant extract	n.d	Increased phagocytic activity	Ghule et al. (2006)
16	Celastraceae	Tripterygium wilfordii Hook.f	Plant extract	n.d	Inhibited phagocytic activity	Chang et al. (1997)
17	Convolvulaceae	Ipomoea batatas (L.) lam	Plant extract	Stimulation of phagosome-lysosome fusion	Increased phagocytic activity	Miyazaki et al. (2005)
18	Convolvulaceae	Cuscuta epithymum (L.) L	Plant extract	Stimulation of syk phosphorylation	Increased phagocytic activity	Sudam et al. (2017)
19	Convolvulaceae	Ipomoea batatas (L.) lam	Plant extract	Stimulation of syk phosphorylation	Increased phagocytic activity	Sudam et al. (2017)
20	Cucurbitaceae	Cucumis maderaspatanus L	Plant extract	Inhibition of reactive oxygen species production	Inhibited phagocytic activity	t Hart et al. (1990)
21	Elaeocarpaceae	Elaeocarpus angustifolius blume	Plant extract	n.d	Increased phagocytic activity	Kakalij et al. (2014)
22	Euphorbiaceae	Euphorbia hirta L	Plant extract	Stimulation of syk phosphorylation	Increased phagocytic activity	Sudam et al. (2017)
23	Fabaceae	Guilandina bonduc L	Plant extract	n.d	Increased phagocytic activity	Shukla et al. (2009)
24	Fabaceae	Vigna mungo (L.) hepper	Plant extract	n.d	Increased phagocytic activity	Solanki and Jain, (2010)
25	Geraniaceae	Geranium sanguineum L	Plant extract	n.d	Increased phagocytic activity	Toshkova et al. (2004)
26	Hypericaceae	Hypericum perforatum L	Plant extract	n.d	Increased phagocytic activity	Fidan et al. (2008)
27	Lamiaceae	Ocimum tenuiflorum L	Plant extract	Stimulation of lysosomal enzymes activity	Increased phagocytic activity	Mukherjee et al. (2005)
28	Lamiaceae	Ajuga reptans L	Plant extract	Inhibition of reactive oxygen species production	Inhibited phagocytic activity	Toiu et al. (2019)
29	Lamiaceae	Ajuga genevensis L	Plant extract	Inhibition of reactive oxygen species production	Inhibited phagocytic activity	Toiu et al. (2019)
30	Lythraceae	Punica granatum L	Plant extract	n.d	No influence on phagocytosis	Oliveira et al. (2010)
31	Malvaceae	Grewia asiatica L	Plant extract	Stimulation of reactive oxygen species production	Increased phagocytic activity	Mesaik et al. (2013)
32	Melastomataceae	Osbeckia octandra DC.	Plant extract	Inhibition of reactive oxygen species production	Inhibited phagocytic activity	t Hart et al. (1990)
33	Meliaceae	Azadirachta indica A.Juss	Plant extract	n.d	Inhibited phagocytic activity	van der Nat et al. (1986)
34	Meliaceae	Melia azedarach L	Plant extract	n.d	No influence on phagocytosis	Benencia et al. (1994)
35	Meliaceae	Cedrela fissilis vell	Plant extract	Inhibition of reactive oxygen species production	Inhibited phagocytic activity	Benencia et al. (1995)
36	Meliaceae	Trichilia glabra L	Plant extract	Inhibition of reactive oxygen species production	Inhibited phagocytic activity	Benencia et al. (2000)
37	Meliaceae	Guarea kunthiana A.Juss	Plant extract	n.d	Increased phagocytic activity	Jerjomiceva et al. (2016)
38	Menispermaceae	Tinospora cordifolia (willd.) Hook.f. and thomson	Plant extract	Stimulation of lysosomal enzymes activity	Increased phagocytic activity	Rege et al. (1993); Mukherjee et al. (2010); Sharma et al. (2012)
39	Menispermaceae	Tinospora crispa (L.) Hook.f. and thomson	Plant extract	Increase of MAC-1 cell surface expression	Increased phagocytic activity	Ahmad et al. (2015)
40	Moraceae	Ficus racemosa L	Plant extract	n.d	Increased phagocytic activity	Heroor et al. (2013)
41	Moringaceae	Moringa oleifera lam	Plant extract	n.d	Increased phagocytic activity	Gupta et al. (2010); Sudha et al. (2010)
42	Phyllanthaceae	Phyllanthus debilis J.G.Klein ex willd	Plant extract	Inhibition of reactive oxygen species production	Inhibited phagocytic activity	t Hart et al. (1990)
43	Phyllanthaceae	Phyllanthus amarus schumach. and thonn	Plant extract	Inhibition of MAC-1 cell surface expression	Inhibited phagocytic activity	Ilangkovan et al. (2015)
44	Poaceae	Saccharum officinarum L	Plant extract	Stimulation of reactive oxygen species production	Increased phagocytic activity	El-Abasy et al. (2002); Lo et al. (2005); Chen et al. (2012)
45	Ranunculaceae	Nigella sativa L	Plant extract	n.d	Inhibited phagocytic activity	Haq et al. (1995)
46	Rosaceae	Crataegus laevigata (poir.) DC.	Plant extract	Inhibition of superoxide anion generation	Inhibited phagocytic activity	Dalli et al. (2008)
47	Rubiaceae	Ixora coccinea L	Plant extract	Inhibition of reactive oxygen species production	Inhibited phagocytic activity	Wickramasinghe et al. (2014)
48	Santalaceae	Viscum album L	Plant extract	n.d	Increased phagocytic activity	Hajto et al. (1989); Fidan et al. (2008)
49	Sapindaceae	Acer pictum subsp. Mono (maxim.) H.Ohashi	Plant extract	Stimulation of reactive oxygen species production	Increased phagocytic activity	An et al. (2013)
50	Saxifragaceae	Bergenia crassifolia (L.) fritsch	Plant extract	Improved neutrophilic uptake capacity	Increased phagocytic activity	Popov et al. (2005)
51	Theaceae	Camellia sinensis (L.) kuntze	Plant extract	Reduction of TLR-4, IkK and CD11 b expression	Inhibited phagocytic activity	Albuquerque et al., (2016)
52	Zingiberaceae	Zingiber zerumbet (L.) roscoe ex sm	Plant extract	Decreased oxidative burst and ROS production	Inhibited phagocytic activity	Akhtar et al. (2019)
53	Zosteraceae	Zostera marina L	Plant extract	n.d	Increased phagocytic activity	Zaporozhets et al. (1991)

n.d.- not defined

Pharmacological modulation of phagocytosis may affect the uptake of pathogens, biochemical changes inside of phago- and lysosomes, phagolysosomes formation and modulation of intracellular killing via oxygen-dependent and oxygen-independent degradation (Table 1).

Among selected plant extracts, two of them, obtained from *Tinospora crispa* (L.) and *Heliotropium sarmentosum* (Lam.), modulated expression of surface receptors. The neutrophils treated with *T. crispa* as well as *H. sarmentosum* extracts overexpressed MAC-1 cell surface receptor leading to increased phagocytic activity (Chen et al., 2014; Ahmad et al., 2015). MAC-1 is a complement receptor (“CR3”) consisting of CD11b (integrin αM) and CD18 (integrin β2) (Todd, 1996). The integrin CD11b is responsible for direct binding to intercellular adhesion molecule-1 (ICAM-1) leading to firm adhesion to endothelium and transmigration of phagocytes to infected tissues (Ross and Vĕtvicka, 1993). Considering that *T. crispa* has been used traditionally in the treatment of rheumatoic arthritis, urinary tract infections, fever, inflammation, fracture, and hypertension (Ahmad et al., 2015), the modulation of phagocytic properties of neutrophils creates new research perspectives for further evaluation. Based on the use in traditional medicine, *H. sarmentosum* has demonstrated anti-inflammatory, antinociceptive and antipyretic activities (Chen et al., 2014), thus similarly to *T. crispa* modulation of phagocytosis may point to unknown immunomodulatory properties of this extract.

Pharmacological stimulation of C3b and Fcγ expression on neutrophil surface was proved for *Echinacea purpurea (L.) Moench* (Melchart et al., 1995; Isaykina et al., 2008). *E. purpurea* has a well-documented biological effect in a variety of diseases, particularly bacterial and viral infections. Recently, many studies are focused on immunomodulatory effects of echinacea. The phytochemical analysis confirmed that alkamides, flavonoids, derivatives of quercetin, kaempferol and caffeic acid, are considered important for biological activity (Barnes et al., 2005).

Popov and colleagues showed that *Bergenia crassifolia* (L.) treatment resulted in stimulation of neutrophilic uptake capacity (Popov et al., 2005). This observation justifies the traditional use of *B. crassifolia* as a treatment of bacterial infections (Shikov et al., 2014). The plant presents a multitude of bioactive agents, including tannins (pedunculagin, tellimagrandin I), polysaccharides, flavonoids (bergenin, kaempferol, and quercetin derivatives) aldehydes (2,4-heptadienal, benzaldehyde), terpenes (thymol, geraniol), phenolics (arbutin, ellagic acid, hydroquinone), phenolic acids (caffeoyl quinic acid, gallic acid, quinic acid) and other compounds (bergenan) (Shikov et al., 2014). The selected compounds are typical for Bergenia species and exerts anti-inflammatory, antimicrobial, antioxidant, and immunomodulating properties (Shikov et al., 2014). A well-studied compound is bergenin, which stimulates the uptake of apoptotic human neutrophils and then intensifies the ROS production by mouse macrophages (Shikov et al., 2014).

*Tinospora cordifolia* (Willd.), *Ocimum tenuiflorum* L. and *Semecarpus anacardium* L. stimulated lysosomal enzymes activity increasing phagocytic properties of neutrophils (Mukherjee et al., 2005; Ramprasath et al., 2006; Sharma et al., 2012). *T. cordifolia* extract was tested on *Escherichia coli*-induced peritonitis in a mice model—both phagocytic and intracellular bactericidal capacities of neutrophils were enhanced by extract treatment (Thatte et al., 1992). The reduction of bacterial colonization in mice model was also observed after *O. tenuiflorum* treatment (Saini et al., 2009).

*Ipomoea batatas* (L.) was identified as an extract capable of stimulating phagolysosome formation inside of neutrophils (Miyazaki et al., 2005).

Inside of newly formed phagolysosomes, ROS are produced more intensively (Nathan, 2006; Urban et al., 2006). One of the major functions of phagocytes is to produce ROS during the oxidative burst (Brungs et al., 2015). Thus, stimulating this process may have beneficial effects during bacterial infections. *Echium amoenum* Fisch (Asadollahi et al., 2015), *Grewia asiatica* L. (Mesaik et al., 2013), *Saccharum officinarum* L. (El-Abasy et al., 2002) and *Acer pictum* subsp. mono (Maxim.) stimulated ROS production and enhanced phagocytosis efficiency. These plants have documented antioxidant and immunomodulatory properties (Abed et al., 2012; Akram et al., 2014; Mehmood et al., 2020).

The signaling pathway leading to ROS release is highly dependent on spleen tyrosine kinase (Syk) activation (Brungs et al., 2015). Three plant extracts (*Cuscuta epithymum* (L.), *Ipomoea batatas* (L.) and *Euphorbia hirta* L.) stimulated Syk phosphorylation resulting in a more pronounced ROS production and an increased phagocytic activity (Sudam et al., 2017).

Besides the plant extracts that stimulated phagocytosis, we also presented plant materials that inhibited this process or had no effect on it (Table 1). As mentioned above, 18 plant extracts decreased phagocytic activity of neutrophils and 2 extracts did not affect this process. Only few molecular mechanisms responsible for suppression of phagocytosis were identified. They were dependent on the inhibition of MAC-1 cell surface expression (*Phyllanthus amarus, Camellia sinensis* (L.)) (Ilangkovan et al., 2015; Albuquerque et al., 2016), suppression of CR1, CR3, CR4, and Fcγ receptors expression (*Areca catechu* L.) (Hung et al., 2006) and inhibition of ROS production (*Cucumis maderaspatanus* L. *Ajuga reptans* L. *Ajuga genevensis* L. *Osbeckia octandra* DC, *Cedrela fissilis* Vell, *Trichilia glabra* L. *Phyllanthus debilis*, *Crataegus laevigata* (Poir.), *Ixora coccinea* L. *Zingiber zerumbet* (L.)) (t Hart et al., 1990; Benencia et al., 1995; Benencia et al., 2000; Dalli et al., 2008; Wickramasinghe et al., 2014; Akhtar et al., 2019; Toiu et al., 2019).

Two plant extracts: *Punica granatum* L. (Oliveira et al., 2010) and *Melia azedarach* L. (Benencia et al., 1994) did not affect phagocytosis. *P. granatum*, is known as a medical plant used for hypertension, diabetes, as well as a few types of cancer, but not for bacteria-induced diseases (Ge et al., 2021). *M. azedarach* was also used for the cancer-related disease treatment (Ervina et al., 2020; Shrestha et al., 2021).

We identified a few 9) plant-derived compounds regulating the neutrophils’ phagocytosis **(** Table 2 **)**. Seven compounds stimulated this process (gingerol, arecoline, 11-hydroxymustakone, N-methyl-2-pyrrolidone, N-formylannonain, magnoflorine, and tinocordiside) (Hung et al., 2006; Sharma et al., 2012; Jin et al., 2016) and two did not affect phagocytosis (cordifolioside A, syringin) (Sharma et al., 2012). Gingerol was isolated from *Pinellia pedatisecta* Schott, arecoline from *Areca catechu* L. and the rest of the selected compounds were isolated from *Tinospora cordifolia* (Willd.). *P. pedatisecta* as well as *A. catechu* are well-known in Chinese medicine as anti-inflammatory agents (Li et al., 2017; Wang et al., 2019). *T. cordifolia* is also known in Asian traditional medicine as an anti-inflammatory agent (Yates et al., 2021).

**TABLE 2:** List of selected compounds isolated from plant material with documented impact on phagocytosis.

	Plant family	Species	Compound	Cellular target and mechanism of action	Effect on phagocytosis	References
1	Araceae	Pinellia pedatisecta schott	Gingerol	n.d	Increased phagocytic activity	Jin et al. (2016)
2	Arecaceae	Areca catechu L	Arecoline	Increased uptake of bacteria	Increased phagocytic activity	Hung et al. (2006)
3	Menispermaceae	Tinospora cordifolia (willd.) Hook.f. and thomson	11-Hydroxymustakone	Increased oxidative burst and ROS production	Increased phagocytic activity	Sharma et al. (2012)
4	Menispermaceae	Tinospora cordifolia (willd.) Hook.f. and thomson	N-methyl-2-pyrrolidone	Increased oxidative burst and ROS production	Increased phagocytic activity	Sharma et al. (2012)
5	Menispermaceae	Tinospora cordifolia (willd.) Hook.f. and thomson	N-formylannonain	Increased oxidative burst and ROS production	Increased phagocytic activity	Sharma et al. (2012)
6	Menispermaceae	Tinospora cordifolia (willd.) Hook.f. and thomson	Cordifolioside A	n.d	No influence on phagocytosis	Sharma et al. (2012)
7	Menispermaceae	Tinospora cordifolia (willd.) Hook.f. and thomson	Magnoflorine	Increased oxidative burst and ROS production	Increased phagocytic activity	Sharma et al. (2012)
8	Menispermaceae	Tinospora cordifolia (willd.) Hook.f. and thomson	Tinocordiside	Increased oxidative burst and ROS production	Increased phagocytic activity	Sharma et al. (2012)
9	Menispermaceae	Tinospora cordifolia (willd.) Hook.f. and thomson	Syringin	n.d	No influence on phagocytosis	Sharma et al. (2012)

n.d.- not defined.

## Pharmacological Modulation of Phagocytosis by Selected Drugs

Some papers reported the effects of synthetic drugs on phagocytic function of neutrophils, but the data are scarce (Table 3). Numerous macrolide antibiotics stimulate phagocytosis performed by macrophages (Hodge et al., 2006; Xu et al., 2019), at least for some of the compounds using phosphatidylserine receptor-dependent pathway (Yamaryo et al., 2003). These results encouraged researchers to test influence of erythromycin (Cuffini et al., 2009) and azithromycin (Pohl et al., 2020) on neutrophil’s phagocytosis, which revealed that the former indeed stimulates phagocytosis, whereas the latter does not affect it. Fosfomycin was shown to enhance intracellular killing of *S. aureus* in both macrophages and neutrophils (Shen et al., 2016). Among antibiotics, chloramphenicol was also tested showing no significant results (Bystrzycka et al., 2017). Clinical significance of those findings remains uncertain, nevertheless testing the influence of other antibiotics, especially macrolides, on phagocytic function should be encouraged.

**TABLE 3:** List of selected synthetic drugs with documented impact on phagocytosis.

	Synthetic drug/s	Cellular target and mechanism of action	Effect on phagocytosis	References
1	Azithromycin	n.d	No influence on phagocytosis	Pohl et al. (2020)
2	Chloramphenicol	n.d	No influence on phagocytosis	Bystrzycka et al. (2017)
3	Erythromycin	n.d	Increased phagocytic activity	Cuffini et al. (2009)
4	Fosfomycin	Increased ROS production and strengthened oxidative burst process	Increased phagocytic activity	Shen et al. (2016)
5	Melatonin	n.d	Decreased phagocytic activity	Xu et al. (2019)

n.d.- not defined.

The interaction between melatonin and immune system is being widely explored, as discussed previously (Carrillo-Vico et al., 2013). Among others, its influence on neutrophil-mediated phagocytosis was tested, showing its inhibitory role in this phenomenon (Xu et al., 2019). The data on beneficial role of melatonin against polymicrobial sepsis also exist (Xu et al., 2019), thus further studies on the exact role of melatonin are needed.

## Pharmacological Modulation of Degranulation via Medical Plants

We have identified 14 plant extracts, whose impact on degranulation was tested (Table 4). All the extracts inhibited the degranulation, but only in 5 cases the mechanism of action was explained. Decreased degranulation resulted from inhibiting the elastase and MPO synthesis and release. An elastase-inhibitory effect was observed after *Typhonium roxburghii* treatment (Korinek et al., 2016), as well as *Panax notoginseng* (Jin et al., 2007), *Lobelia chinensis Lour* (Kuo et al., 2011) and *Tamarindus indica* L. (Paula et al., 2009). Selected plants were widely used to treat cancer and inflammatory-related diseases (Jin et al., 2007; Kuo et al., 2011; Korinek et al., 2016).

**TABLE 4:** List of selected plant material with documented impact on degranulation of neutrophils.

	Plant family	Species	Plant material	Cellular target and mechanism of action	Effect on degranulation	References
1	Apiaceae	Cnidium monnieri (L.) cusson	Plant extract	n.d	Decreased degranulation	Park et al. (2004)
2	Apiaceae	Angelica gigas nakai	Plant extract	n.d	Decreased degranulation	Park et al. (2004)
3	Araceae	Typhonium roxburghii schott	Plant extract	Inhibition of elastase release	Decreased degranulation	Korinek et al. (2016)
4	Araliaceae	Panax notoginseng (burkill) F.H.Chen	Plant extract	Inhibition of elastase release	Decreased degranulation	Jin et al. (2007)
5	Asteraceae	Carthamus tinctorius L	Plant extract	n.d	Decreased degranulation	Park et al. (2004)
6	Asteraceae	Anvillea garcinii subsp. Radiata (coss. and durieu) anderb	Plant extract	Inhibition of myeloperoxidase (MPO) release	Decreased degranulation	Boukemara et al. (2016)
7	Campanulaceae	Lobelia chinensis lour	Plant extract	Inhibition of elastase release	Decreased degranulation	Kuo et al. (2011)
8	Fabaceae	Tamarindus indica L	Plant extract	Inhibition of elastase release	Decreased degranulation	Paula et al. (2009)
9	Lauraceae	Cinnamomum cassia (L.) J.Presl	Plant extract	n.d	Decreased degranulation	Park et al. (2004)
10	Meliaceae	Guarea kunthiana A.Juss	Plant extract	n.d	Decreased degranulation	Jerjomiceva et al. (2016)
11	Myrtaceae	Eugenia aurata	Plant extract	n.d	Decreased degranulation	Costa et al. (2016)
12	Myrtaceae	Eugenia punicifolia	Plant extract	n.d	Decreased degranulation	Costa et al. (2016)
13	Orobanchaceae	Rehmannia glutinosa (gaertn.) DC.	Plant extract	n.d	Decreased degranulation	Park et al. (2004)
14	Rosaceae	Prunus persica (L.) batsch	Plant extract	n.d	Decreased degranulation	Park et al. (2004)

n.d.- not defined.

Anti-MPO activity was observed for *Anvillea garcinii* subsp. radiate (Boukemara et al., 2016). This plant was used for symptomatic treatment of cold (Perveen et al., 2020), but the successful treatment was related to the presence of flavonoids and sesquiterpene lactones, which show significant antibacterial and antifungal properties (Perveen et al., 2020). Thus, based on traditional use of *A. garcinii*, further evaluation of phagocytosis seems to be worth exploring.

For the rest of tested extracts (*Cnidium monnieri* (L.), *Angelica gigas* Nakai, *Carthamus* tinctorius L. *Cinnamomum cassia* (L.), *Guarea kunthiana* A. Juss. *Prunus persica* (L.), *Rehmannia glutinosa* (Gaertn.), *Eugenia aurata*, and *Eugenia punicifolia*) authors documented only final effect on degranulation without identifying the molecular basis of the observed effect (Park et al., 2004; Costa et al., 2016; Jerjomiceva et al., 2016).

Chung and colleagues identified *Hypericum geminiflorum* Hemsl. as a source of compounds modulating degranulation (Table 5). Gemichalcone A and gemichalcone B. isolated from *H. geminiflorum* decreased the degranulation of neutrophils (Chung et al., 2002). Plants belonging to the Hypericum species were used as diuretics, analgetics, antiphlogistics and antidepressant agents (Zhang et al., 2020a). Immunomodulatory, anti-inflammatory or antimicrobial properties of Hypericium species are unknown. Another example of a plant-derived compound decreasing degranulation of neutrophils is quercetin (Kanashiro et al., 2007; Pečivová et al., 2012). Although quercetin is widespread in many plant species, in both mentioned articles quercetin was used as a synthesized compound. Quercetin is a plant molecule that has shown many pharmacological activities, such as antimicrobial, antiviral, anticancer, having potential for treating metabolic, allergic, and inflammatory disorders, cardiovascular diseases, and arthritis (Batiha et al., 2020). The documented pharmacological effects encouraged to introduction of quercetin to the pharmaceutical market, however, low solubility in water, which is a key factor in drug absorption and its bioavailability, limits its use (Batiha et al., 2020). Further attempts to increase the quercetin solubility are urgently needed. Interestingly, quercetin, as well as described above gemichalcone A and gemichalcone B belong to flavonoids (Panche et al., 2016), which may indicate a specific chemical group of plant-derived compounds that modulate neutrophil degranulation.

**TABLE 5:** List of selected compounds isolated from plant material with documented impact on degranulation of neutrophils.

	Plant family	Species	Compound	Cellular target and mechanism of action	Effect on degranulation	References
1	Hypericaceae	Hypericum geminiflorum hemsl	Gemichalcone A	n.d	Decreased degranulation	Chung et al. (2002)
2	Hypericaceae	Hypericum geminiflorum hemsl	Gemichalcone B	n.d	Decreased degranulation	Chung et al. (2002)
3	Lamiaceae	Salvia officinalis L	Salvianolic acid	n.d	Increased degranulation	Wang et al. (2010)

n.d.- not defined.

Another source of compounds modulating degranulation is *Salvia officinalis* L.–it contains salvianolic acid capable to stimulate degranulation (Wang et al., 2010). Although mentioned data refer to mast cells the observed pharmacological effect is noteworthy, and in our opinion further studies on neutrophil model may also bring promising results *S. officinalis* was used for the treatment of inflammation, gout, paralysis, ulcers, rheumatoid arthritis, dizziness, tremor, diarrhea, and hyperglycemia (Ghorbani and Esmaeilizadeh, 2017). Salvianolic acid is considered a chemo-preventive agent which suppresses oxidative stress and apoptosis (Wang et al., 2021). It was also documented that salvianolic acid may also be an antioxidant and anti-inflammatory agent which modulates PI3K/Akt/mTOR signaling pathway (Jin et al., 2021).

## Pharmacological Modulation of Degranulation via Drugs

Number of synthetic drugs known to regulate neutrophils degranulation is even lower than that affecting phagocytosis (Table 6). Similarly, two macrolide antibiotics were tested, and it was shown that erythromycin stimulates degranulation (Abdelghaffar et al., 1997), whereas azithromycin does not affect this process (Pohl et al., 2020). Chloramphenicol was also investigated, but exerted no effect on this function (Bystrzycka et al., 2017). Again, whether these effects are of clinical significance, remains unclear.

**TABLE 6:** List of selected synthetic drugs with documented impact on degranulation of neutrophils.

	Synthetic drug/s	Cellular target and mechanism of action	Effect on degranulation	References
1	Azithromycin	n.d	No influence on degranulation	Pohl et al. (2020)
2	Chloramphenicol	n.d	No influence on degranulation	Bystrzycka et al. (2017)
3	Erythromycin	Decreased oxidative burst and ROS production	Increased degranulation	Abdelghaffar et al. (1997)

n.d.- not defined.

## Pharmacological Modulation of NETosis via Medical Plants

A few plant extracts were tested for their influence on NETosis (Table 7), showing that extracts obtained from *Anacardium occidentale* L. (Hollands et al., 2016), *Verbesina erstediana* Benth (Yaseen et al., 2017), and *Guarea kunthiana* A. Juss (Jerjomiceva et al., 2016) increased it, whereas *Salvia miltiorrhiza* Bunge (Tao et al., 2018), *Eugenia aurata* (Costa et al., 2016), and *Eugenia punicifolia* (Costa et al., 2016) decreased it.

**TABLE 7:** List of selected plant material with documented impact on NET formation.

	Plant family	Species	Plant extract/s or compound/s	Cellular target and mechanism of action	Effect on NET formation	References
1	Anacardiaceae	Anacardium occidentale L	Plant extract	Activation of PI3 kinase and surface-expressed G protein-coupled sphingosine-1-phosphate (S1P) receptors	Increased NET formation	Hollands et al. (2016)
2	Asteraceae	Verbesina erstediana benth	Plant extract	n.d	Increased NET formation	Yaseen et al. (2017)
3	Lamiaceae	Salvia miltiorrhiza bunge	Plant extract	Inhibition of myeloperoxidase (MPO) and NADPH oxidase (NOX) activity	Decreased NET formation	Tao et al. (2018)
4	Meliaceae	Guarea kunthiana A.Juss	Plant extract	n.d	Increased NET formation	Jerjomiceva et al. (2016)
5	Myrtaceae	Eugenia aurata	Plant extract	n.d	Decreased NET formation	Costa et al. (2016)
6	Myrtaceae	Eugenia punicifolia	Plant extract	n.d	Decreased NET formation	Costa et al. (2016)

n.d.- not defined.

*A. occidentale* has shown significant antibacterial activity and might have potential applications as new antibacterial drug (Shabeeba and Rathinasamy, 2018). *A. occidentale* extract has been tested in mice and rat models as a therapy of gastric-related conditions (Konan and Bacchi, 2007; Morais et al., 2010; Goulart da Silva et al., 2021). Thus, further evaluation of its immunomodulatory properties may open novel prospects. *V. erstediana* and *G. kunthiana*were were used in the folk medicine in treatment of bacterial infections, especially *S. aureus* (Jerjomiceva et al., 2016; Yaseen et al., 2017). Thus, documented antimicrobial properties justify the traditional use.

It is unknown why *E. aurata* and *E. punicifolia* extracts decreased NET release. For *S. miltiorrhiza* it has been speculated that decreased NET formation may results from the inhibition of MPO and NADPH oxidase (NOX) activity (Tao et al., 2018).

Six plant-derived compounds were investigated (Table 8). Anacardic acid, as the only substance, was able to increase NET production (Hollands et al., 2016), whereas catechin hydrate, epicatechin, rutin trihydrate (Kirchner et al., 2013), celastrol (Yu et al., 2015), and polydatin (Liao et al., 2018) decreased it. Anacardic acid belongs to phenolic lipids (Hollands et al., 2016), a diversified group of compounds derived from mono and dihydroxyphenols (Stasiuk and Kozubek, 2010). Anacardic acid shows antioxidant capacity, what may be related with suppression of superoxide generation (Trevisan et al., 2006). According to Hollands et al. inhibition of ROS production (inter alia superoxide generation) directly affects NET formation (Hollands et al., 2016).

**TABLE 8:** List of selected compounds isolated from plant material with documented impact on NET formation.

	Plant-derived compound	Cellular target and mechanism of action	Effect on NET formation	References
1	Anacardic acid	Activation of PI3 kinase and surface-expressed G protein-coupled sphingosine-1-phosphate (S1P) receptors	Increased NET formation	Hollands et al. (2016)
2	Catechin hydrate	Decreased ROS production	Decreased NET formation	Kirchner et al. (2013)
3	Celastrol	Downregulation the SYK-mek-erk-nf?b signaling cascade	Decreased NET formation	Yu et al. (2015)
4	Epicatechin	Decreased ROS production	Decreased NET formation	Kirchner et al. (2013)
5	Polydatin	Decreased ROS production	Decreased NET formation	Liao et al. (2018)
6	Rutin trihydrate	Decreased ROS production	Decreased NET formation	Kirchner et al. (2013)

n.d.- not defined.

Other compounds (catechin hydrate, epicatechin, rutin trihydrate, celastrol, and polydatin) classified as NET formation inhibitors belong to different chemical groups, thus an unambiguous assignment of a chemical structure to a biological function is not possible. Interestingly, excluding celastrol, compounds decreased NET formation via modulation of ROS production. Although anacardic acid also decreased ROS production, the biological effect was opposite to the one observed for other compounds. Because ROS production is crucial for efficient pathogen killing and NET formation, the dependency between chemical structure and pharmacological effect, as well as potential cytotoxicity need to be evaluated in the future.

## Pharmacological Modulation of NET Formation via Drugs

We identified 19 drugs, whose impact on NET formation was tested, mostly antibiotics and antimycotics (Table 9). β-lactams belong to the most widely used class of antibiotics (Bush and Bradford, 2016), but only two drugs from this group were tested. Amoxicillin increased NET formation (Bystrzycka et al., 2016), whereas no such effect was observed for cefotaxime (Manda-Handzlik et al., 2017). Three representatives of macrolides were investigated with different results. Clarithromycin increased NETosis (Konstantinidis et al., 2016; Arampatzioglou et al., 2018), erythromycin decreased it (Zhang et al., 2019), whereas azithromycin exerted no effect (Manda-Handzlik et al., 2017). Among other groups single particles were tested. Enrofloxacin (fluoroquinolone used in veterinary medicine (López-Cadenas et al., 2013)) increased NET formation (Jerjomiceva et al., 2014), chloramphenicol (Bystrzycka et al., 2017) and gentamicin (Manda-Handzlik et al., 2017) decreased it, whereas minocycline (tetracycline used in acne vulgaris, but with some safety issues, as revised in (Garner et al., 2012)) did not affect this effect (Irizarry-Caro et al., 2018).

**TABLE 9:** List of selected synthetic drugs with documented impact on NET formation.

	Synthetic drug/s	Cellular target and mechanism of action	Effect on NET formation	References
1	Amoxicillin	n.d	Increased NET formation	Bystrzycka et al. (2016)
2	Amphotericin B	n.d	Decreased NET formation	Decker et al. (2018)
3	Azithromycin	n.d	Decreased NET formation	Bystrzycka et al. (2017)
4	Cefotaxime	n.d	No effect on NET formation	Manda-Handzlik et al. (2017)
5	Clarithromycin	n.d	Increased NET formation	Konstantinidis et al. (2016); Arampatzioglou et al. (2018)
6	Chloramphenicol	n.d	Decreased NET formation	Bystrzycka et al. (2017)
7	Clozapine	n.d	No effect on NET formation	Irizarry-Caro et al. (2018)
8	Enrofloxacin	Increased expression of PAD (peptidylarginine deiminase) 4 protein, increased presence of citrullinated H3	Increased NET formation	Jerjomiceva et al. (2014)
9	Erythromycin	n.d	Decreased NET formation	Zhang et al. (2019)
10	17-β-estradiol	n.d	Increased NET formation	Yasuda et al. (2019)
11	Gentamicin	n.d	Decreased NET formation	Manda-Handzlik et al. (2017)
12	Hydralazine	n.d	Increased NET formation	Irizarry-Caro et al. (2018)
13	Mannitol hypertonic saline	Suppresses NOX2-dependent NETosis is via neutrophil dehydration	Decreased NET formation	Nadesalingam et al. (2018)
14	Melatonin	n.d	Increased NET formation	Xu et al. (2019)
15	Memantine	Stimulates the production of MPO	Increased NET formation	Peng et al. (2020)
16	Metformin	Reduction of elastase, proteinase-3 and histones concentration	Decreased NET formation	Menegazzo et al. (2018)
17	Minocycline	n.d	No effect on NET formation	Irizarry-Caro et al. (2018)
18	Procainamide	n.d	Increased NET formation	Irizarry-Caro et al. (2018)
19	Voriconazole	n.d	Decreased NET formation	Decker et al. (2018)

n.d.- not defined.

Neutrophils play an essential role in counteracting mycoses, including NET release, as recently revised (Urban and Nett, 2019). Both amphotericin B, usually used in severe cases (Hamill, 2013), and voriconazole, a drug of choice in invasive aspergillosis (Malani et al., 2015), decreased NET formation (Decker et al., 2018).

Above-mentioned data clearly reveal the complexity of the issue. It obvious that stimulation of NET production during an infection is beneficial, as it increases pathogen clearance and thus infection control, on the other hand it can participate in host tissue’s damage, as it is observed in acute respiratory distress syndrome (Grégoire et al., 2018), or even promote bacteria proliferation (Czaikoski et al., 2016). The effects of antimicrobial drugs are not easy to interpret due to difficulties in separating direct antimicrobial and immune-modulating properties. To further investigate this matter, *in vivo* studies on combinations of antibiotics or antimycotics with NET-modulating particles are needed. The substances discussed below can be used as such particles.

It was demonstrated that acidification inhibits ROS-dependent NET release (Behnen et al., 2017), whereas more alkaline pH stimulates NET formation mediated by both NOX2-independent (Naffah de Souza et al., 2017) and NOX2-dependent (Khan et al., 2018) pathways. These results are of interest since hypoperfusion-induced lactic acidemia is common in sepsis and septic shock and should be managed accordingly (Rhodes et al., 2017). It is worth verifying whether increasing pH affects NET release during the treatment.

Mannitol and hypertonic saline, agents administered to patients with intracranial edema (Burgess et al., 2016) may suppress of NOX2-dependent NET production (Nadesalingam et al., 2018). Again, the clinical significance of this observation is not clear.

Metformin remains the first line of pharmacological treatment in type 2 diabetes mellitus (Baker et al., 2021) and may be beneficial in numerous other conditions (Lv and Guo, 2020). Studies showed that it decreases NET release through inhibition of elastase, proteinase-3 and histones (Menegazzo et al., 2018). Clinical significance of this phenomenon is not clear, nevertheless it should be noted, that in sepsis and septic shock accompanied with hyperglycemia insulin is a drug of choice (Rhodes et al., 2017). As mentioned, NET formation seems to play a role in atherosclerosis (Soehnlein, 2012), which may be prevented by a long-term metformin treatment (as revised in (Luo et al., 2019)).

Melatonin was also tested for its effect on NET production and increased it (Xu et al., 2019). Among other hormones only 17-β-estradiol was tested–it also increased NET formation (Yasuda et al., 2019). Since women have overall stronger immune responses, as revised in (Moulton, 2018), further studies are of interest.

Memantine is a drug approved for Alzheimer’s disease and tested for other applications (Lu and Nasrallah, 2018). It was shown that through stimulation of MPO production, it increases NET formation (Peng et al., 2020). Clozapine, the first atypical antipsychotic drug, whose side effects involve agranulocytosis (Khokhar et al., 2018) was also tested and was shown to exert no influence on NET formation (Irizarry-Caro et al., 2018).

Drug-induced lupus is a condition similar to systemic lupus erythematosus and triggered by initiation of a certain drug, usually resolving after the medication’s withdrawal (Borchers et al., 2007). Carmona-Rivera et al. demonstrated a role of NETosis in this pathology (Carmona-Rivera et al., 2017). In this context, an interesting approach was proposed by (Irizarry-Caro et al., 2018), who focused on drugs related to drug-induced lupus. They investigated hydralazin, a direct-acting vasodilator (Kandler et al., 2011) and procainamide, and showed that both drugs indeed increase NET production, which may contribute to autoimmunity.

## Conclusion and Prospects

The Chinese and European Pharmacopeia convincingly report possible modulations of the immune response via medical plants. So far, natural products classified as immunomodulators were cursorily explored, without comprehensive identification of molecular mechanisms responsible for the observed effects. Firstly, there is a need to perform detailed phytochemical analyses of plant extracts, then select pure compounds for further biological evaluation. A specific assessment of purified compounds could help to identify cellular targets, determine bioactive dosage, biodistribution and kinetics. The knowledge of molecular mechanisms regulated by plant-derived compounds may potentially help to identify therapeutic targets, as well as potentially limit the spread of an infection. Moreover, the chemical structures of selected secondary metabolites can serve as lead structures for synthesis of new substances. Similar conclusions could be drawn for synthetic drugs. Only few synthetic drugs were tested for their influence on immunomodulatory neutrophil functions and most of the data brought interesting results, thus further studies are urgently needed. For synthetic drugs, two areas seem to be the most important: identifying new substances, which affect effector functions of neutrophils, and testing approved drugs, especially antibiotics, antimycotics and these responsible for autoimmune reactions re, for their impact on immunomodulatory functions of neutrophils.

## References

[B1] Abdelghaffar H. Vazifeh D. Labro M. T. (1997). Erythromycin A-Derived Macrolides Modify the Functional Activities of Human Neutrophils by Altering the Phospholipase D-Phosphatidate Phosphohydrolase Transduction Pathway: L-Cladinose Is Involved Both in Alterations of Neutrophil Functions and Modulation of This Transductional Pathway. J. Immunol. 159, 3995–4005. 9378989

[B2] Abed A. Minaiyan M. Ghannadi A. Mahzouni P. Babavalian M. R. (2012). Effect of *Echium* amoenum Fisch. et Mey a Traditional Iranian Herbal Remedy in an Experimental Model of Acute Pancreatitis. ISRN Gastroenterol. 2012, 141548. 10.5402/2012/141548 23008778 PMC3449129

[B3] Adrover J. M. Nicolás-Ávila J. A. Hidalgo A. (2016). Aging: A Temporal Dimension for Neutrophils. Trends Immunol. 37, 334–345. 10.1016/j.it.2016.03.005 27083489

[B4] Ahmad W. Jantan I. Kumolosasi E. Bukhari S. N. (2015). Immunostimulatory Effects of the Standardized Extract of Tinospora Crispa on Innate Immune Responses in Wistar Kyoto Rats. Drug Des. Devel Ther. 9, 2961–2973. 10.2147/DDDT.S85405 PMC4468953 26089645

[B5] Akhtar N. M. Y. Jantan I. Arshad L. Haque M. A. (2019). Standardized Ethanol Extract, Essential Oil and Zerumbone of Zingiber Zerumbet Rhizome Suppress Phagocytic Activity of Human Neutrophils. BMC Complement. Altern. Med. 19, 331. 10.1186/s12906-019-2748-5 31752812 PMC6873536

[B6] Akram M. Hamid A. Khalil A. Ghaffar A. Tayyaba N. Saeed A. (2014). Review on Medicinal Uses, Pharmacological, Phytochemistry and Immunomodulatory Activity of Plants. Int. J. Immunopathol Pharmacol. 27, 313–319. 10.1177/039463201402700301 25280022

[B7] Albuquerque K. F. F. S. Marinovic M. P. Morandi A. C. Bolin A. P. Otton R. (2016). Green Tea Polyphenol Extract In Vivo Attenuates Inflammatory Features of Neutrophils from Obese Rats. Eur. J. Nutr. 55, 1261–1274. 10.1007/s00394-015-0940-z 26031433

[B8] An B.-S. Kang J.-H. Yang H. Yang M.-P. Jeung E.-B. (2013). Effects of Acer Okamotoanum Sap on the Function of Polymorphonuclear Neutrophilic Leukocytes In Vitro and In Vivo. Mol. Med. Rep. 7, 654–658. 10.3892/mmr.2012.1190 23165961

[B9] Angus D. C. Linde-Zwirble W. T. Lidicker J. Clermont G. Carcillo J. Pinsky M. R. (2001). Epidemiology of Severe Sepsis in the United States: Analysis of Incidence, Outcome, and Associated Costs of Care. Crit. Care Med. 29, 1303–1310. 10.1097/00003246-200107000-00002 11445675

[B10] Anzai A. Choi J. L. He S. Fenn A. M. Nairz M. Rattik S. (2017). The Infarcted Myocardium Solicits GM-CSF for the Detrimental Oversupply of Inflammatory Leukocytes. J. Exp. Med. 214, 3293–3310. 10.1084/jem.20170689 28978634 PMC5679174

[B11] Arampatzioglou A. Papazoglou D. Konstantinidis T. Chrysanthopoulou A. Mitsios A. Angelidou I. (2018). Clarithromycin Enhances the Antibacterial Activity and Wound Healing Capacity in Type 2 Diabetes Mellitus by Increasing LL-37 Load on Neutrophil Extracellular Traps. Front. Immunol. 9, 2064. 10.3389/fimmu.2018.02064 30250474 PMC6139320

[B12] Arcanjo A. Logullo J. Menezes C. C. B. De Souza Carvalho Giangiarulo T. C. Dos Reis M. C. De Castro G. M. M. (2020). The Emerging Role of Neutrophil Extracellular Traps in Severe Acute Respiratory Syndrome Coronavirus 2 (COVID-19). Sci. Rep. 10, 19630. 10.1038/s41598-020-76781-0 33184506 PMC7665044

[B13] Asadollahi F. Mehrzad J. Chaichi M. J. Taghavi Razavizadeh A. (2015). Photoimmunological Properties of Borage in Bovine Neutrophil In Vitro Model. J. Photochem. Photobiol. B: Biol. 151, 270–275. 10.1016/j.jphotobiol.2015.08.023 26334939

[B14] Athens J. W. Haab O. P. Raab S. O. Mauer A. M. Ashenbrucker H. Cartwright G. E. (1961a). Leukokinetic Studies. Iv. The Total Blood, Circulating and Marginal Granulocyte Pools and the Granulocyte Turnover Rate in Normal Subjects*. J. Clin. Invest. 40, 989–995. 10.1172/jci104338 13684958 PMC290816

[B15] Athens J. W. Raab S. O. Haab O. P. Mauer A. M. Ashenbrucker H. Cartwright G. E. (1961b). Leukokinetic Studies. Iii. The Distribution of Granulocytes in the Blood of Normal Subjects*. J. Clin. Invest. 40, 159–164. 10.1172/jci104230 13684959 PMC290701

[B16] Baker C. Retzik-Stahr C. Singh V. Plomondon R. Anderson V. Rasouli N. (2021). Should Metformin Remain the First-Line Therapy for Treatment of Type 2 Diabetes?. Ther. Adv. Endocrinol. Metab. 12, 2042018820980225. 10.1177/2042018820980225 33489086 PMC7809522

[B17] Barnes J. Anderson L. A. Gibbons S. Phillipson J. D. (2005). Echinacea Species (Echinacea Angustifolia (DC.) Hell., Echinacea Pallida (Nutt.) Nutt.,Echinacea Purpurea (L.) Moench): a Review of Their Chemistry, Pharmacology and Clinical Properties. J. Pharm. Pharmacol. 57, 929–954. 10.1211/0022357056127 16102249

[B18] Batiha G. E. Beshbishy A. M. Ikram M. Mulla Z. S. El-Hack M. E. A. Taha A. E. (2020). The Pharmacological Activity, Biochemical Properties, and Pharmacokinetics of the Major Natural Polyphenolic Flavonoid: Quercetin. Foods 9, 374. 10.3390/foods9030374 PMC7143931 32210182

[B19] Behl T. Kumar K. Brisc C. Rus M. Nistor-Cseppento D. C. Bustea C. (2021). Exploring the Multifocal Role of Phytochemicals as Immunomodulators. Biomed. Pharmacother. 133, 110959. 10.1016/j.biopha.2020.110959 33197758

[B20] Behnen M. Möller S. Brozek A. Klinger M. Laskay T. (2017). Extracellular Acidification Inhibits the ROS-dependent Formation of Neutrophil Extracellular Traps. Front. Immunol. 8, 184. 10.3389/fimmu.2017.00184 28293240 PMC5329032

[B21] Benencia F. Courrèges M. C. Massouh E. J. Coulombié F. C. (1994). Effect of Melia Azedarach L. Leaf Extracts on Human Complement and Polymorphonuclear Leukocytes. J. Ethnopharmacol. 41, 53–57. 10.1016/0378-8741(94)90057-4 8170159

[B22] Benencia F. Courrèges M. C. Nores M. M. Coulombié F. C. (1995). Immunomodulatory Activities of Cedrela Tubiflora Leaf Aqueous Extracts. J. Ethnopharmacol. 49, 133–139. 10.1016/0378-8741(95)01311-3 8824738

[B23] Benencia F. Courrèges M. C. Coulombié F. C. (2000). Anti-inflammatory Activities of Trichilia Glabra Aqueous Leaf Extract. J. Ethnopharmacol. 71, 293–300. 10.1016/s0378-8741(00)00192-6 10904176

[B24] Benjamim C. F. Hogaboam C. M. Kunkel S. L. (2004). The Chronic Consequences of Severe Sepsis. J. Leukoc. Biol. 75, 408–412. 10.1189/jlb.0503214 14557384

[B25] Blaisdell A. Crequer A. Columbus D. Daikoku T. Mittal K. Dey S. K. (2015). Neutrophils Oppose Uterine Epithelial Carcinogenesis via Debridement of Hypoxic Tumor Cells. Cancer Cell 28, 785–799. 10.1016/j.ccell.2015.11.005 26678340 PMC4698345

[B26] Borchers A. T. Keen C. L. Gershwin M. E. (2007). Drug-induced Lupus. Ann. N Y Acad. Sci. 1108, 166–182. 10.1196/annals.1422.019 17893983

[B27] Borregaard N. (2010). Neutrophils, from Marrow to Microbes. Immunity 33, 657–670. 10.1016/j.immuni.2010.11.011 21094463

[B28] Boudoukha C. Bouriche H. Ortega E. Senator A. (2016). Immunomodulatory Effects ofSantolina Chamaecyparissusleaf Extracts on Human Neutrophil Functions. Pharm. Biol. 54, 667–673. 10.3109/13880209.2015.1071853 26453376

[B29] Boukemara H. Hurtado-Nedelec M. Marzaioli V. Bendjeddou D. El Benna J. Marie J. C. (2016). Anvillea Garcinii Extract Inhibits the Oxidative Burst of Primary Human Neutrophils. BMC Complement. Altern. Med. 16, 433. 10.1186/s12906-016-1411-7 27809835 PMC5095960

[B30] Brinkmann V. Reichard U. Goosmann C. Fauler B. Uhlemann Y. Weiss D. S. (2004). Neutrophil Extracellular Traps Kill Bacteria. Science 303, 1532–1535. 10.1126/science.1092385 15001782

[B31] Brungs S. Kolanus W. Hemmersbach R. (2015). Syk Phosphorylation—a Gravisensitive Step in Macrophage Signalling. Cell Commun. Signaling 13, 9. 10.1186/s12964-015-0088-8 PMC4326470 25644261

[B32] Buckley C. D. Ross E. A. Mcgettrick H. M. Osborne C. E. Haworth O. Schmutz C. (2006). Identification of a Phenotypically and Functionally Distinct Population of Long-Lived Neutrophils in a Model of Reverse Endothelial Migration. J. Leukoc. Biol. 79, 303–311. 10.1189/jlb.0905496 16330528

[B33] Buckley C. D. Gilroy D. W. Serhan C. N. (2014). Proresolving Lipid Mediators and Mechanisms in the Resolution of Acute Inflammation. Immunity 40, 315–327. 10.1016/j.immuni.2014.02.009 24656045 PMC4004957

[B34] Burgess S. Abu-Laban R. B. Slavik R. S. Vu E. N. Zed P. J. (2016). A Systematic Review of Randomized Controlled Trials Comparing Hypertonic Sodium Solutions and Mannitol for Traumatic Brain Injury. Ann. Pharmacother. 50, 291–300. 10.1177/1060028016628893 26825644

[B35] Bush K. Bradford P. A. (2016). β-Lactams and β-Lactamase Inhibitors: An Overview. Cold Spring Harb Perspect. Med. 6. 10.1101/cshperspect.a025247 PMC4968164 27329032

[B36] Bystrzycka W. Moskalik A. Sieczkowska S. Manda-Handzlik A. Demkow U. Ciepiela O. (2016). The Effect of Clindamycin and Amoxicillin on Neutrophil Extracellular Trap (NET) Release. cejoi 1, 1–5. 10.5114/ceji.2016.58811 PMC4829816 27095915

[B37] Bystrzycka W. Manda-Handzlik A. Sieczkowska S. Moskalik A. Demkow U. Ciepiela O. (2017). Azithromycin and Chloramphenicol Diminish Neutrophil Extracellular Traps (NETs) Release. Int. J. Mol. Sci. 18, 2666. 10.3390/ijms18122666 PMC5751268 29292737

[B38] Carmona-Rivera C. Purmalek M. M. Moore E. Waldman M. Walter P. J. Garraffo H. M. (2017). A Role for Muscarinic Receptors in Neutrophil Extracellular Trap Formation and Levamisole-Induced Autoimmunity. JCI Insight 2, e89780. 10.1172/jci.insight.89780 28194438 PMC5291726

[B39] Carrillo-Vico A. Lardone P. Álvarez-Sánchez N. Rodríguez-Rodríguez A. Guerrero J. (2013). Melatonin: Buffering the Immune System. Ijms 14, 8638–8683. 10.3390/ijms14048638 23609496 PMC3645767

[B40] Casanova-Acebes M. Pitaval C. Weiss L. A. Nombela-Arrieta C. Chèvre R. A-González N. (2013). Rhythmic Modulation of the Hematopoietic Niche through Neutrophil Clearance. Cell 153, 1025–1035. 10.1016/j.cell.2013.04.040 23706740 PMC4128329

[B41] Chang D. M. Chang W. Y. Kuo S. Y. Chang M. L. (1997). The Effects of Traditional Antirheumatic Herbal Medicines on Immune Response Cells. J. Rheumatol. 24, 436–441. 9058645

[B42] Chen M.-H. Lo D.-Y. Liao J.-W. Hsuan S.-L. Chien M.-S. Lin C.-C. (2012). Immunostimulation of Sugar Cane Extract on Neutrophils to Salmonella typhimurium Infection in Mice. Phytother. Res. 26, 1062–1067. 10.1002/ptr.3676 22213156

[B43] Chen M.-L. Wu S. Tsai T.-C. Wang L.-K. Chou W.-M. Tsai F.-M. (2014). The Caffeic Acid in Aqueous Extract ofTournefortia Sarmentosaenhances Neutrophil Phagocytosis ofEscherichia Coli. Immunopharmacol. Immunotoxicol. 36, 390–396. 10.3109/08923973.2014.956753 25311172

[B44] Chousterman B. G. Swirski F. K. Weber G. F. (2017). Cytokine Storm and Sepsis Disease Pathogenesis. Semin. Immunopathol 39, 517–528. 10.1007/s00281-017-0639-8 28555385

[B45] Chung M.-I. Weng J.-R. Wang J.-P. Teng C.-M. Lin C.-N. (2002). Antiplatelet and Anti-inflammatory Constituents and New Oxygenated Xanthones from *Hypericum* Geminiflorum. Planta Med. 68, 25–29. 10.1055/s-2002-19871 11842322

[B46] Coffelt S. B. Kersten K. Doornebal C. W. Weiden J. Vrijland K. Hau C.-S. (2015). IL-17-producing γδ T Cells and Neutrophils Conspire to Promote Breast Cancer Metastasis. Nature 522, 345–348. 10.1038/nature14282 25822788 PMC4475637

[B47] Colom B. Bodkin J. V. Beyrau M. Woodfin A. Ody C. Rourke C. (2015). Leukotriene B4-Neutrophil Elastase *Axis* Drives Neutrophil Reverse Transendothelial Cell Migration *In Vivo* . Immunity 42, 1075–1086. 10.1016/j.immuni.2015.05.010 26047922 PMC4504024

[B48] Colotta F. Re F. Polentarutti N. Sozzani S. Mantovani A. (1992). Modulation of Granulocyte Survival and Programmed Cell Death by Cytokines and Bacterial Products. Blood 80, 2012–2020. 10.1182/blood.v80.8.2012.2012 1382715

[B49] Costa M. F. Jesus T. I. Lopes B. R. Angolini C. F. Montagnolli A. Gomes L. P. (2016). Eugenia Aurata and Eugenia Punicifolia HBK Inhibit Inflammatory Response by Reducing Neutrophil Adhesion, Degranulation and NET Release. BMC Complement. Altern. Med. 16, 403. 10.1186/s12906-016-1375-7 27770779 PMC5075401

[B50] Csepregi J. Z. Orosz A. Zajta E. Kása O. Németh T. Simon E. (2018). Myeloid-Specific Deletion of Mcl-1 Yields Severely Neutropenic Mice that Survive and Breed in Homozygous Form. J.I. 201, 3793–3803. 10.4049/jimmunol.1701803 PMC6287103 30464050

[B51] Cuffini A. M. Tullio V. Banche G. Allizond V. Mandras N. Roana J. (2009). The Erythromycin-Resistance inS. PyogenesDoes Not Limit the Human Polymorphonuclear Cell Antimicrobial Activity. Int. J. Immunopathol Pharmacol. 22, 239–242. 10.1177/039463200902200127 19309572

[B52] Czaikoski P. G. Mota J. M. Nascimento D. C. Sônego F. Castanheira F. V. Melo P. H. (2016). Neutrophil Extracellular Traps Induce Organ Damage during Experimental and Clinical Sepsis. PLoS One 11, e0148142. 10.1371/journal.pone.0148142 26849138 PMC4743982

[B53] Czerwińska M. E. Melzig M. F. (2018). Cornus Mas and Cornus Officinalis-Analogies and Differences of Two Medicinal Plants Traditionally Used. Front. Pharmacol. 9, 894. 10.3389/fphar.2018.00894 30210335 PMC6121078

[B54] Dalli E. Milara J. Cortijo J. Morcillo E. Cosinsales J. Sotillo J. (2008). Hawthorn Extract Inhibits Human Isolated Neutrophil Functions. Pharmacol. Res. 57, 445–450. 10.1016/j.phrs.2008.05.001 18547815

[B55] De Santo C. Arscott R. Booth S. Karydis I. Jones M. Asher R. (2010). Invariant NKT Cells Modulate the Suppressive Activity of IL-10-secreting Neutrophils Differentiated with Serum Amyloid A. Nat. Immunol. 11, 1039–1046. 10.1038/ni.1942 20890286 PMC3001335

[B56] Decker C. Wurster S. Lazariotou M. Hellmann A.-M. Einsele H. Ullmann A. J. (2018). Analysis of the In Vitro Activity of Human Neutrophils against Aspergillus fumigatus in Presence of Antifungal and Immunosuppressive Agents. Med. Mycol. 56, 514–519. 10.1093/mmy/myx069 29420763

[B57] Deniset J. F. Kubes P. (2018). Neutrophil Heterogeneity: Bona Fide Subsets or Polarization States? J. Leukoc. Biol. 103, 829–838. 10.1002/jlb.3ri0917-361r 29462505

[B58] Deniset J. F. Surewaard B. G. Lee W.-Y. Kubes P. (2017). Splenic Ly6Ghigh Mature and Ly6Gint Immature Neutrophils Contribute to Eradication of S. Pneumoniae. J. Exp. Med. 214, 1333–1350. 10.1084/jem.20161621 28424248 PMC5413339

[B59] Eash K. J. Means J. M. White D. W. Link D. C. (2009). CXCR4 Is a Key Regulator of Neutrophil Release from the Bone Marrow under Basal and Stress Granulopoiesis Conditions. Blood 113, 4711–4719. 10.1182/blood-2008-09-177287 19264920 PMC2680371

[B60] Eichelberger K. R. Goldman W. E. (2020). Manipulating Neutrophil Degranulation as a Bacterial Virulence Strategy. Plos Pathog. 16, e1009054. 10.1371/journal.ppat.1009054 33301542 PMC7728292

[B61] El-Abasy M. Motobu M. Shimura K. Na K.-J. Kang C.-B. Koge K. (2002). Immunostimulating and Growth-Promoting Effects of Sugar Cane Extract (SCE) in Chickens. J. Vet. Med. Sci. 64, 1061–1063. 10.1292/jvms.64.1061 12499696

[B62] Ella K. Mócsai A. Káldi K. (2018). Circadian Regulation of Neutrophils: Control by a Cell-Autonomous Clock or Systemic Factors? Eur. J. Clin. Invest. 48 (Suppl. 2), e12965. 10.1111/eci.12965 29877596

[B63] Eltzschig H. K. Eckle T. (2011). Ischemia and Reperfusion-From Mechanism to Translation. Nat. Med. 17, 1391–1401. 10.1038/nm.2507 22064429 PMC3886192

[B64] Ervina M. Poerwono H. Widyowati R. Matsunami K. Sukardiman (2020). Bio-selective Hormonal Breast Cancer Cytotoxic and Antioxidant Potencies of Melia Azedarach L. Wild Type Leaves. Biotechnol. Rep. (Amst) 25, e00437. 10.1016/j.btre.2020.e00437 32140442 PMC7044715

[B65] Erwig L. P. Kluth D. C. Walsh G. M. Rees A. J. (1998). Initial Cytokine Exposure Determines Function of Macrophages and Renders Them Unresponsive to Other Cytokines. J. Immunol. 161, 1983–1988. 9712070

[B66] Evrard M. Kwok I. W. H. Chong S. Z. Teng K. W. W. Becht E. Chen J. (2018). Developmental Analysis of Bone Marrow Neutrophils Reveals Populations Specialized in Expansion, Trafficking, and Effector Functions. Immunity 48, 364–379. 10.1016/j.immuni.2018.02.002 29466759

[B67] Fairn G. D. Grinstein S. (2012). How Nascent Phagosomes Mature to Become Phagolysosomes. Trends Immunol. 33, 397–405. 10.1016/j.it.2012.03.003 22560866

[B68] Faix J. D. (2013). Biomarkers of Sepsis. Crit. Rev. Clin. Lab. Sci. 50, 23–36. 10.3109/10408363.2013.764490 23480440 PMC3613962

[B69] Faurschou M. Borregaard N. (2003). Neutrophil Granules and Secretory Vesicles in Inflammation. Microbes Infect. 5, 1317–1327. 10.1016/j.micinf.2003.09.008 14613775

[B70] Fidan I. Ozkan S. Gurbuz I. Yesilyurt E. Erdal B. Yolbakan S. (2008). The Efficiency ofViscum Album Ssp. albumandHypericum Perforatumon Human Immune CellsIn Vitro. Immunopharmacol. Immunotoxicol. 30, 519–528. 10.1080/08923970802135286 18668395

[B71] Figueiredo-Rinhel A. S. G. De Melo L. L. Bortot L. O. Santos E. O. L. Andrade M. F. Azzolini A. E. C. S. (2017). Baccharis Dracunculifolia DC (Asteraceae) Selectively Modulates the Effector Functions of Human Neutrophils. J. Pharm. Pharmacol. 69, 1829–1845. 10.1111/jphp.12822 28994118

[B72] Fridlender Z. G. Sun J. Kim S. Kapoor V. Cheng G. Ling L. (2009). Polarization of Tumor-Associated Neutrophil Phenotype by TGF-β: "N1" versus "N2" TAN. Cancer Cell 16, 183–194. 10.1016/j.ccr.2009.06.017 19732719 PMC2754404

[B73] Gabrilovich D. I. (2017). Myeloid-Derived Suppressor Cells. Cancer Immunol. Res. 5, 3–8. 10.1158/2326-6066.cir-16-0297 28052991 PMC5426480

[B74] Galluzzi L. Vitale I. Aaronson S. A. Abrams J. M. Adam D. Agostinis P. (2018). Molecular Mechanisms of Cell Death: Recommendations of the Nomenclature Committee on Cell Death 2018. Cell Death Differ 25, 486–541. 10.1038/s41418-017-0012-4 29362479 PMC5864239

[B75] Garcia-Romo G. S. Caielli S. Vega B. Connolly J. Allantaz F. Xu Z. (2011). Netting Neutrophils Are Major Inducers of Type I IFN Production in Pediatric Systemic Lupus Erythematosus. Sci. Transl Med. 3, 73ra20. 10.1126/scitranslmed.3001201 PMC3143837 21389264

[B76] Garner C. V. D'amico R. Simms H. H. (1996). Cytokine-mediated Human Polymorphonuclear Neutrophil Phagocytosis: Evidence of Differential Sensitivities to Manipulation of Intracellular Mechanisms. J. Surg. Res. 60, 84–90. 10.1006/jsre.1996.0015 8592438

[B77] Garner S. E. Eady A. Bennett C. Newton J. N. Thomas K. Popescu C. M. (2012). Minocycline for Acne Vulgaris: Efficacy and Safety. Cochrane Database Syst. Rev. 15. Cd002086. 10.1002/14651858.cd002086.pub2 PMC7017847 22895927

[B78] Ge S. Duo L. Wang J. Gegen Z. Yang J. Li Z. (2021). A Unique Understanding of Traditional Medicine of Pomegranate, Punica Granatum L. And its Current Research Status. J. Ethnopharmacol 10, 113877. 10.1016/j.jep.2021.113877 33515685

[B79] Gentles A. J. Newman A. M. Liu C. L. Bratman S. V. Feng W. Kim D. (2015). The Prognostic Landscape of Genes and Infiltrating Immune Cells across Human Cancers. Nat. Med. 21, 938–945. 10.1038/nm.3909 26193342 PMC4852857

[B80] Ghorbani A. Esmaeilizadeh M. (2017). Pharmacological Properties of Salvia Officinalis and its Components. J. Traditional Complement. Med. 7, 433–440. 10.1016/j.jtcme.2016.12.014 PMC5634728 29034191

[B81] Ghule B. V. Murugananthan G. Nakhat P. D. Yeole P. G. (2006). Immunostimulant Effects of *Capparis* Zeylanica Linn. Leaves. J. Ethnopharmacol. 108, 311–315. 10.1016/j.jep.2006.03.041 16766150

[B82] Gierlikowska B. Gierlikowski W. Bekier K. Skalicka-Woźniak K. Czerwińska M. E. Kiss A. K. (2020a). Inula Helenium and Grindelia Squarrosa as a Source of Compounds with Anti-inflammatory Activity in Human Neutrophils and Cultured Human Respiratory Epithelium. J. Ethnopharmacol, 249, 112311. 10.1016/j.jep.2019.112311 31644941

[B83] Gierlikowska B. Gierlikowski W. Demkow U. (2020b). Alantolactone Enhances the Phagocytic Properties of Human Macrophages and Modulates Their Proinflammatory Functions. Front. Pharmacol. 11, 1339. 10.3389/fphar.2020.01339 33013371 PMC7494907

[B84] Goulart Da Silva G. De Oliveira Braga L. E. Souza De Oliveira E. C. Valério Tinti S. De Carvalho J. E. Goldoni Lazarini J. (2021). Cashew Apple Byproduct: Gastroprotective Effects of Standardized Extract. J. Ethnopharmacol. 269, 113744. 10.1016/j.jep.2020.113744 33359862

[B85] Granot Z. Henke E. Comen E. A. King T. A. Norton L. Benezra R. (2011). Tumor Entrained Neutrophils Inhibit Seeding in the Premetastatic Lung. Cancer Cell 20, 300–314. 10.1016/j.ccr.2011.08.012 21907922 PMC3172582

[B86] Grégoire M. Uhel F. Lesouhaitier M. Gacouin A. Guirriec M. Mourcin F. (2018). Impaired Efferocytosis and Neutrophil Extracellular Trap Clearance by Macrophages in ARDS. Eur. Respir. J. 52, 1702590. 10.1183/13993003.02590-2017 29946009

[B87] Grigore A. Cord D. Tanase C. Albulescu R. (2020). Herbal Medicine, a Reliable Support in COVID Therapy. J. Immunoassay Immunochemistry 41, 976–999. 10.1080/15321819.2020.1862867 33356860

[B88] Grommes J. Soehnlein O. (2011). Contribution of Neutrophils to Acute Lung Injury. Mol. Med. 17, 293–307. 10.2119/molmed.2010.00138 21046059 PMC3060975

[B89] Gupta A. Gautam M. K. Singh R. K. Kumar M. V. Rao Ch V. Goel R. K. (2010). Immunomodulatory Effect of Moringa Oleifera Lam. Extract on Cyclophosphamide Induced Toxicity in Mice. Indian J. Exp. Biol. 48, 1157–1160. 21117458

[B90] Hacbarth E. Kajdacsy-Balla A. (1986). Low Density Neutrophils in Patients with Systemic Lupus Erythematosus, Rheumatoid Arthritis, and Acute Rheumatic Fever. Arthritis Rheum. 29, 1334–1342. 10.1002/art.1780291105 2430586

[B91] Hajto T. Hostanska K. Gabius H. J. (1989). Modulatory Potency of the Beta-galactoside-specific Lectin from Mistletoe Extract (Iscador) on the Host Defense System In Vivo in Rabbits and Patients. Cancer Res. 49, 4803–4808. 2758413

[B92] Hamill R. J. (2013). Amphotericin B Formulations: a Comparative Review of Efficacy and Toxicity. Drugs 73, 919–934. 10.1007/s40265-013-0069-4 23729001

[B93] Haq A. Abdullatif M. Lobo P. I. Khabar K. S. A. Sheth K. V. Al-Sedairy S. T. (1995). Nigella Sativa: Effect on Human Lymphocytes and Polymorphonuclear Leukocyte Phagocytic Activity. Immunopharmacol, 30, 147–155. 10.1016/0162-3109(95)00016-m 8530256

[B94] He W. Holtkamp S. Hergenhan S. M. Kraus K. De Juan A. Weber J. (2018). Circadian Expression of Migratory Factors Establishes Lineage-specific Signatures that Guide the Homing of Leukocyte Subsets to Tissues. Immunity 49, 1175–1190. 10.1016/j.immuni.2018.10.007 30527911 PMC6303219

[B95] Heroor S. Beknal A. Mahurkar N. (2013). Immunomodulatory Activity of Methanolic Extracts of Fruits and Bark of *Ficus* Glomerata Roxb. In Mice and on Human Neutrophils. Indian J. Pharmacol. 45, 130–135. 10.4103/0253-7613.108287 23716887 PMC3660923

[B96] Hodge S. Hodge G. Brozyna S. Jersmann H. Holmes M. Reynolds P. N. (2006). Azithromycin Increases Phagocytosis of Apoptotic Bronchial Epithelial Cells by Alveolar Macrophages. Eur. Respir. J. 28, 486–495. 10.1183/09031936.06.00001506 16737992

[B97] Hollands A. Corriden R. Gysler G. Dahesh S. Olson J. Raza Ali S. (2016). Natural Product Anacardic Acid from Cashew Nut Shells Stimulates Neutrophil Extracellular Trap Production and Bactericidal Activity. J. Biol. Chem. 291, 13964–13973. 10.1074/jbc.m115.695866 27226531 PMC4933157

[B98] Hong C. Kidani Y. A-Gonzalez N. Phung T. Ito A. Rong X. (2012). Coordinate Regulation of Neutrophil Homeostasis by Liver X Receptors in Mice. J. Clin. Invest. 122, 337–347. 10.1172/jci58393 22156197 PMC3248291

[B99] Horby P. Horby P. Lim W. S. Emberson J. R. Mafham M. Bell J. L. (2021). Dexamethasone in Hospitalized Patients with Covid-19. N. Engl. J. Med. 384, 693–704. 10.1056/NEJMoa2021436 32678530 PMC7383595

[B100] Houghton A. M. Rzymkiewicz D. M. Ji H. Gregory A. D. Egea E. E. Metz H. E. (2010). Neutrophil Elastase-Mediated Degradation of IRS-1 Accelerates Lung Tumor Growth. Nat. Med. 16, 219–223. 10.1038/nm.2084 20081861 PMC2821801

[B101] Hu S. Concha C. Cooray R. Holmberg O. (1995). Ginseng-enhanced Oxidative and Phagocytic Activities of Polymorphonuclear Leucocytes from Bovine Peripheral Blood and Stripping Milk. Vet. Res. 26, 155–161. 7795665

[B102] Hung S.-L. Lee Y.-Y. Liu T.-Y. Peng J.-L. Cheng Y.-Y. Chen Y.-T. (2006). Modulation of Phagocytosis, Chemotaxis, and Adhesion of Neutrophils by Areca Nut Extracts. J. Periodontol. 77, 579–585. 10.1902/jop.2006.050217 16584337

[B103] Ilangkovan M. Jantan I. Mesaik M. A. Bukhari S. N. (2015). Immunosuppressive Effects of the Standardized Extract of *Phyllanthus* Amarus on Cellular Immune Responses in Wistar-Kyoto Rats. Drug Des. Devel Ther. 9, 4917–4930. 10.2147/DDDT.S88189 PMC4555964 26347462

[B104] Ionita M. G. Van Den Borne P. Catanzariti L. M. Moll F. L. De Vries J.-P. P. M. Pasterkamp G. (2010). High Neutrophil Numbers in Human Carotid Atherosclerotic Plaques Are Associated with Characteristics of Rupture-Prone Lesions. Atvb 30, 1842–1848. 10.1161/atvbaha.110.209296 20595650

[B105] Irizarry-Caro J. A. Carmona-Rivera C. Schwartz D. M. Khaznadar S. S. Kaplan M. J. Grayson P. C. (2018). Brief Report: Drugs Implicated in Systemic Autoimmunity Modulate Neutrophil Extracellular Trap Formation. Arthritis Rheumatol. 70, 468–474. 10.1002/art.40372 29121457 PMC5826843

[B106] Isaykina N. V. Perevozchicova T. V. Kalinkina G. I. (2008). Immunotropic Activity of Plant Extract Echinosol. Bull. Exp. Biol. Med. 146, 223–225. 10.1007/s10517-008-0257-5 19145323

[B107] Jaumouillé V. Waterman C. M. (2020). Physical Constraints and Forces Involved in Phagocytosis. Front. Immunol. 11, 1097. 10.3389/fimmu.2020.01097 32595635 PMC7304309

[B108] Jenne C. N. Wong C. H. Y. Zemp F. J. Mcdonald B. Rahman M. M. Forsyth P. A. (2013). Neutrophils Recruited to Sites of Infection Protect from Virus Challenge by Releasing Neutrophil Extracellular Traps. Cell Host & Microbe 13, 169–180. 10.1016/j.chom.2013.01.005 23414757

[B109] Jerjomiceva N. Seri H. Völlger L. Wang Y. Zeitouni N. Naim H. Y. (2014). Enrofloxacin Enhances the Formation of Neutrophil Extracellular Traps in Bovine Granulocytes. J. Innate Immun. 6, 706–712. 10.1159/000358881 24642685 PMC4140967

[B110] Jerjomiceva N. Seri H. Yaseen R. De Buhr N. Setzer W. N. Naim H. Y. (2016). Guarea Kunthiana Bark Extract Enhances the Antimicrobial Activities of Human and Bovine Neutrophils. Nat. Prod. Commun. 11, 767–770. 10.1177/1934578x1601100617 27534112

[B111] Jin U.-H. Park S.-G. Suh S.-J. Kim J.-K. Kim D.-S. Moon S.-K. (2007). Inhibitory Effect ofPanax Notoginseng on Nitric Oxide Synthase, Cyclo-Oxygenase-2 and Neutrophil Functions. Phytother. Res. 21, 142–148. 10.1002/ptr.2018 17128437

[B112] Jin Y. P. Wu H. Yu H. L. Pan Y. Z. Chen Y. Q. Wang K. L. (2016). [Antagonism Mechanism of Gingerols against Inflammatory Effect of Toxic Raphides from Pinella Pedatisecta]. Zhongguo Zhong Yao Za Zhi. 41, 1087–1092. 10.4268/cjcmm20160619 28875675

[B113] Jin L. Chen C. Huang L. Bu L. Zhang L. Yang Q. (2021). Salvianolic Acid A Blocks Vasculogenic Mimicry Formation in Human Non-small Cell Lung Cancer via PI3K/Akt/mTOR Signalling. Clin. Exp. Pharmacol. Physiol. 48, 508–514. 10.1111/1440-1681.13464 33529404

[B114] Johansson A. Jesaitis A. J. Lundqvist H. Magnusson K.-E. Sjölin C. Karlsson A. (1995). Different Subcellular Localization of Cytochrome B and the Dormant NADPH-Oxidase in Neutrophils and Macrophages: Effect on the Production of Reactive Oxygen Species during Phagocytosis. Cell Immunol. 161, 61–71. 10.1006/cimm.1995.1009 7867086

[B115] Kakalij R. M. Alla C. P. Kshirsagar R. P. Kumar B. H. Mutha S. S. Diwan P. V. (2014). Ameliorative Effect of *Elaeocarpus* Ganitrus on Gentamicin-Induced Nephrotoxicity in Rats. Indian J. Pharmacol. 46, 298–302. 10.4103/0253-7613.132163 24987177 PMC4071707

[B116] Kanashiro A. Souza J. G. Kabeya L. M. C. S. Azzolini A. E. Lucisano-Valim Y. M. (2007). Elastase Release by Stimulated Neutrophils Inhibited by Flavonoids: Importance of the Catechol Group. Z. Naturforsch C J. Biosci. 62, 357–361. 10.1515/znc-2007-5-607 17708440

[B117] Kandler M. R. Mah G. T. Tejani A. M. Stabler S. N. Salzwedel D. M. (2011). Hydralazine for Essential Hypertension. Cochrane Database Syst. Rev. 9, Cd004934. 10.1002/14651858.cd004934.pub4 PMC13158553 22071816

[B118] Kessenbrock K. Krumbholz M. Schönermarck U. Back W. Gross W. L. Werb Z. (2009). Netting Neutrophils in Autoimmune Small-Vessel Vasculitis. Nat. Med. 15, 623–625. 10.1038/nm.1959 19448636 PMC2760083

[B119] Khan M. A. Philip L. M. Cheung G. Vadakepeedika S. Grasemann H. Sweezey N. (2018). Regulating NETosis: Increasing pH Promotes NADPH Oxidase-dependent NETosis. Front. Med. (Lausanne) 5, 19. 10.3389/fmed.2018.00019 29487850 PMC5816902

[B120] Khandpur R. Carmona-Rivera C. Vivekanandan-Giri A. Gizinski A. Yalavarthi S. Knight J. S. (2013). NETs Are a Source of Citrullinated Autoantigens and Stimulate Inflammatory Responses in Rheumatoid Arthritis. Sci. Transl Med. 5, 178ra140. 10.1126/scitranslmed.3005580 PMC3727661 23536012

[B121] Khokhar J. Y. Henricks A. M. Sullivan E. D. K. Green A. I. (2018). Unique Effects of Clozapine: A Pharmacological Perspective. Adv. Pharmacol. 82, 137–162. 10.1016/bs.apha.2017.09.009 29413518 PMC7197512

[B122] Kim M.-H. Granick J. L. Kwok C. Walker N. J. Borjesson D. L. Curry F.-R. E. (2011). Neutrophil Survival and C-Kit+-Progenitor Proliferation in *Staphylococcus* Aureus-Infected Skin Wounds Promote Resolution. Blood 117, 3343–3352. 10.1182/blood-2010-07-296970 21278352 PMC3069674

[B123] Kirchner T. Hermann E. Möller S. Klinger M. Solbach W. Laskay T. (2013). Flavonoids and 5-aminosalicylic Acid Inhibit the Formation of Neutrophil Extracellular Traps. Mediators Inflamm. 2013, 710239. 10.1155/2013/710239 24381411 PMC3871909

[B124] Kiss A. K. Michalak B. Patyra A. Majdan M. (2020). UHPLC‐DAD‐ESI‐MS/MS and HPTLC Profiling of Ash Leaf Samples from Different Commercial and Natural Sources and Their In Vitro Effects on Mediators of Inflammation. Phytochem. Anal. 31, 57–67. 10.1002/pca.2866 31286597

[B125] Klopf J. Brostjan C. Eilenberg W. Neumayer C. (2021). Neutrophil Extracellular Traps and Their Implications in Cardiovascular and Inflammatory Disease. Int. J. Mol. Sci. 22, 559. 10.3390/ijms22020559 PMC7828090 33429925

[B126] Kolaczkowska E. Kubes P. (2013). Neutrophil Recruitment and Function in Health and Inflammation. Nat. Rev. Immunol. 13, 159–175. 10.1038/nri3399 23435331

[B127] Konan N. A. Bacchi E. M. (2007). Antiulcerogenic Effect and Acute Toxicity of a Hydroethanolic Extract from the Cashew (Anacardium Occidentale L.) Leaves. J. Ethnopharmacol 112, 237–242. 10.1016/j.jep.2007.03.003 17499463

[B128] Konstantinidis T. Kambas K. Mitsios A. Panopoulou M. Tsironidou V. Dellaporta E. (2016). Immunomodulatory Role of Clarithromycin in Acinetobacter Baumannii Infection via Formation of Neutrophil Extracellular Traps. Antimicrob. Agents Chemother. 60, 1040–1048. 10.1128/aac.02063-15 26643338 PMC4750671

[B129] Korinek M. Chen K.-M. Jiang Y.-H. El-Shazly M. Stocker J. Chou C.-K. (2016). Anti-allergic Potential of *Typhonium* Blumei: Inhibition of Degranulation via Suppression of PI3K/PLCγ2 Phosphorylation and Calcium Influx. Phytomedicine 23, 1706–1715. 10.1016/j.phymed.2016.10.011 27912872

[B130] Kreisel D. Nava R. G. Li W. Zinselmeyer B. H. Wang B. Lai J. (2010). *In vivo* two-photon Imaging Reveals Monocyte-dependent Neutrophil Extravasation during Pulmonary Inflammation. Proc. Natl. Acad. Sci. 107, 18073–18078. 10.1073/pnas.1008737107 20923880 PMC2964224

[B131] Krishnamoorthy N. Douda D. N. Brüggemann T. R. Ricklefs I. Duvall M. G. Abdulnour R. E. (2018). Neutrophil Cytoplasts Induce T(H)17 Differentiation and Skew Inflammation toward Neutrophilia in Severe Asthma. Sci. Immunol. 3. 10.1126/sciimmunol.aao4747 PMC6320225 30076281

[B132] Kuo P.-C. Hwang T.-L. Lin Y.-T. Kuo Y.-C. Leu Y.-L. (2011). Chemical Constituents from *Lobelia* Chinensis and Their Anti-virus and Anti-inflammatory Bioactivities. Arch. Pharm. Res. 34, 715–722. 10.1007/s12272-011-0503-7 21656355

[B133] Küster H. Weiss M. Willeitner A. E. Detlefsen S. Jeremias I. Zbojan J. (1998). Interleukin-1 Receptor Antagonist and Interleukin-6 for Early Diagnosis of Neonatal Sepsis 2 Days before Clinical Manifestation. Lancet. 352, 1271–1277. 10.1016/s0140-6736(98)08148-3 9788457

[B134] Lande R. Ganguly D. Facchinetti V. Frasca L. Conrad C. Gregorio J. (2011). Neutrophils Activate Plasmacytoid Dendritic Cells by Releasing Self-DNA-Peptide Complexes in Systemic Lupus Erythematosus. Sci. Translational Med. 3, 73ra19. 10.1126/scitranslmed.3001180 PMC3399524 21389263

[B135] Lee Y.-Y. Lin M.-B. Cheng C.-F. Chang L.-Y. Liu T.-Y. Hung S.-L. (2014). Inhibitory Effects of Areca Nut Extract on Expression of Complement Receptors and Fc Receptors in Human Neutrophils. J. Periodontol. 85, 1096–1106. 10.1902/jop.2013.130498 24354650

[B136] Lee K. H. Kronbichler A. Park D. D.-Y. Park Y. Moon H. Kim H. (2017). Neutrophil Extracellular Traps (NETs) in Autoimmune Diseases: A Comprehensive Review. Autoimmun. Rev. 16, 1160–1173. 10.1016/j.autrev.2017.09.012 28899799

[B137] Ley K. Laudanna C. Cybulsky M. I. Nourshargh S. (2007). Getting to the Site of Inflammation: the Leukocyte Adhesion Cascade Updated. Nat. Rev. Immunol. 7, 678–689. 10.1038/nri2156 17717539

[B138] Li L. Luo Z. Liu Y. Wang H. Liu A. Yu G. (2017). Screening and Identification of the Metabolites in Rat Plasma and Urine after Oral Administration of Areca Catechu L. Nut Extract by Ultra-high-pressure Liquid Chromatography Coupled with Linear Ion Trap-Orbitrap Tandem Mass Spectrometry. Molecules 22, 1026. 10.3390/molecules22061026 PMC6152711 28635656

[B139] Liao P. He Y. Yang F. Luo G. Zhuang J. Zhai Z. (2018). Polydatin Effectively Attenuates Disease Activity in Lupus-Prone Mouse Models by Blocking ROS-Mediated NET Formation. Arthritis Res. Ther. 20, 254. 10.1186/s13075-018-1749-y 30419963 PMC6235205

[B140] Licciardi P. V. Underwood J. R. (2011). Plant-derived Medicines: a Novel Class of Immunological Adjuvants. Int. Immunopharmacology 11, 390–398. 10.1016/j.intimp.2010.10.014 21056709

[B141] Lien D. C. Wagner W. W. Jr. Capen R. L. Haslett C. Hanson W. L. Hofmeister S. E. (1987). Physiological Neutrophil Sequestration in the Lung: Visual Evidence for Localization in Capillaries. J. Appl. Physiol. 62, 1236–1243. 10.1152/jappl.1987.62.3.1236 3106311

[B142] Lieschke G. Grail D. Hodgson G. Metcalf D. Stanley E. Cheers C. (1994). Mice Lacking Granulocyte Colony-Stimulating Factor Have Chronic Neutropenia, Granulocyte and Macrophage Progenitor Cell Deficiency, and Impaired Neutrophil Mobilization. Blood 84, 1737–1746. 10.1182/blood.v84.6.1737.bloodjournal8461737 7521686

[B143] Liew P. X. Kubes P. (2019). The Neutrophil's Role during Health and Disease. Physiol. Rev. 99, 1223–1248. 10.1152/physrev.00012.2018 30758246

[B144] Lo D.-Y. Chen T.-H. Chien M.-S. Koge K. Hosono A. Kaminogawa S. (2005). Effects of Sugar Cane Extract on the Modulation of Immunity in Pigs. J. Vet. Med. Sci. 67, 591–597. 10.1292/jvms.67.591 15997186

[B145] López-Cadenas C. Sierra-Vega M. García-Vieitez J. Diez-Liébana M. Sahagún-Prieto A. Fernández- Martínez N. (2013). Enrofloxacin: Pharmacokinetics and Metabolism in Domestic Animal Species. Cdm 14, 1042–1058. 10.2174/1389200214666131118234935 24261706

[B146] Lu S. Nasrallah H. A. (2018). The Use of Memantine in Neuropsychiatric Disorders: An Overview. Ann. Clin. Psychiatry 30, 234–248. 30028898

[B147] Luo F. Das A. Chen J. Wu P. Li X. Fang Z. (2019). Metformin in Patients with and without Diabetes: a Paradigm Shift in Cardiovascular Disease Management. Cardiovasc. Diabetol. 18, 54. 10.1186/s12933-019-0860-y 31029144 PMC6486984

[B148] Lv Z. Guo Y. (2020). Metformin and its Benefits for Various Diseases. Front. Endocrinol. (Lausanne) 11, 191. 10.3389/fendo.2020.00191 32425881 PMC7212476

[B149] Malani A. N. Kerr L. E. Kauffman C. A. (2015). Voriconazole: How to Use This Antifungal Agent and what to Expect. Semin. Respir. Crit. Care Med. 36, 786–795. 10.1055/s-0035-1562903 26398543

[B150] Manda A. Pruchniak M. P. Araźna M. Demkow U. A. (2014). Neutrophil Extracellular Traps in Physiology and Pathology. cejoi 1, 116–121. 10.5114/ceji.2014.42136 PMC4439979 26155111

[B151] Manda-Handzlik A. Bystrzycka W. Sieczkowska S. Demkow U. Ciepiela O. (2017). Antibiotics Modulate the Ability of Neutrophils to Release Neutrophil Extracellular Traps. Adv. Exp. Med. Biol. 944, 47–52. 10.1007/978-3-319-44488-8_59 27826884

[B152] Mantovani A. Cassatella M. A. Costantini C. Jaillon S. (2011). Neutrophils in the Activation and Regulation of Innate and Adaptive Immunity. Nat. Rev. Immunol. 11, 519–531. 10.1038/nri3024 21785456

[B153] Martin G. S. Mannino D. M. Eaton S. Moss M. (2003). The Epidemiology of Sepsis in the United States from 1979 through 2000. N. Engl. J. Med. 348, 1546–1554. 10.1056/nejmoa022139 12700374

[B154] Marty C. Misset B. Tamion F. Fitting C. Carlet J. Cavaillon J.-M. (1994). Circulating Interleukin-8 Concentrations in Patients with Multiple Organ Failure of Septic and Nonseptic Origin. Crit. Care Med. 22, 673–679. 10.1097/00003246-199404000-00025 8143477

[B155] Mauer A. M. Athens J. W. Ashenbrucker H. Cartwright G. E. Wintrobe M. M. (1960). Leukokinetic Studies. Ii. A Method for Labeling Granulocytes In Vitro with Radioactive Diisopropylfluorophosphate (Dfp32)*. J. Clin. Invest. 39, 1481–1486. 10.1172/jci104167 16695832 PMC293394

[B156] Mcmillan R. Scott J. L. (1968). Leukocyte Labeling with 51Chromium. Blood 32, 738–754. 10.1182/blood.v32.5.738.738 5687937

[B157] Mehmood A. Ishaq M. Usman M. Zhao L. Ullah A. Wang C. (2020). Nutraceutical Perspectives and Value Addition of Phalsa ( Grewia Asiatica L.): A Review. J. Food Biochem. 44, e13228. 10.1111/jfbc.13228 32320069

[B158] Meijer M. Rijkers G. T. Van Overveld F. J. (2013). Neutrophils and Emerging Targets for Treatment in Chronic Obstructive Pulmonary Disease. Expert Rev. Clin. Immunol. 9, 1055–1068. 10.1586/1744666x.2013.851347 24168412

[B159] Melchart D. Linde K. Worku F. Sarkady L. Holzmann M. Jurcic K. (1995). Results of Five Randomized Studies on the Immunomodulatory Activity of Preparations of Echinacea. J. Altern. Complement. Med. 1, 145–160. 10.1089/acm.1995.1.145 9395611

[B160] Menegazzo L. Scattolini V. Cappellari R. Bonora B. M. Albiero M. Bortolozzi M. (2018). The Antidiabetic Drug Metformin Blunts NETosis In Vitro and Reduces Circulating NETosis Biomarkers In Vivo. Acta Diabetol. 55, 593–601. 10.1007/s00592-018-1129-8 29546579

[B161] Mesaik M. A. Ahmed A. Khalid A. S. Jan S. Siddiqui A. A. Perveen S. (2013). Effect of Grewia Asiatica Fruit on Glycemic Index and Phagocytosis Tested in Healthy Human Subjects. Pak J. Pharm. Sci. 26, 85–89. 23261731

[B162] Mitsios A. Arampatzioglou A. Arelaki S. Mitroulis I. Ritis K. (2016). NETopathies? Unraveling the Dark Side of Old Diseases through Neutrophils. Front. Immunol. 7, 678. 10.3389/fimmu.2016.00678 28123386 PMC5225098

[B163] Miyazaki Y. Kusano S. Doi H. Aki O. (2005). Effects on Immune Response of Antidiabetic Ingredients from White-Skinned Sweet Potato (*Ipomoea* Batatas L.). Nutrition 21, 358–362. 10.1016/j.nut.2004.11.001 15797679

[B164] Morais T. C. Pinto N. B. Carvalho K. M. M. B. Rios J. B. Ricardo N. M. P. S. Trevisan M. T. S. (2010). Protective Effect of Anacardic Acids from Cashew (Anacardium Occidentale) on Ethanol-Induced Gastric Damage in Mice. Chem. Biol. Interact. 183, 264–269. 10.1016/j.cbi.2009.10.008 19853593

[B165] Mortaz E. Alipoor S. D. Adcock I. M. Mumby S. Koenderman L. (2018). Update on Neutrophil Function in Severe Inflammation. Front. Immunol. 9, 2171. 10.3389/fimmu.2018.02171 30356867 PMC6190891

[B166] Mostafa E. Fayed M. A. A. Radwan R. A. Bakr R. O. (2019). Centaurea Pumilio L. Extract and Nanoparticles: A Candidate for Healthy Skin. Colloids Surf. B: Biointerfaces 182, 110350. 10.1016/j.colsurfb.2019.110350 31326622

[B167] Mottola G. (2014). The Complexity of Rab5 to Rab7 Transition Guarantees Specificity of Pathogen Subversion Mechanisms. Front Cel Infect Microbiol. 4, 180. 10.3389/fcimb.2014.00180 PMC4273659 25566515

[B168] Moulton V. R. (2018). Sex Hormones in Acquired Immunity and Autoimmune Disease. Front. Immunol. 9, 2279. 10.3389/fimmu.2018.02279 30337927 PMC6180207

[B169] Mukherjee R. Dash P. K. Ram G. C. (2005). Immunotherapeutic Potential of Ocimum Sanctum (L) in Bovine Subclinical Mastitis. Res. Vet. Sci. 79, 37–43. 10.1016/j.rvsc.2004.11.001 15894022

[B170] Mukherjee R. De U. K. Ram G. C. (2010). Evaluation of Mammary Gland Immunity and Therapeutic Potential of Tinospora Cordifolia against Bovine Subclinical Mastitis. Trop. Anim. Health Prod. 42, 645–651. 10.1007/s11250-009-9471-z 19876755

[B171] Nadesalingam A. Chen J. H. K. Farahvash A. Khan M. A. (2018). Hypertonic Saline Suppresses NADPH Oxidase-dependent Neutrophil Extracellular Trap Formation and Promotes Apoptosis. Front. Immunol. 9, 359. 10.3389/fimmu.2018.00359 29593709 PMC5859219

[B172] Naffah de Souza C. Breda L. C. D. Khan M. A. De Almeida S. R. Câmara N. O. S. Sweezey N. (2017). Alkaline pH Promotes NADPH Oxidase-independent Neutrophil Extracellular Trap Formation: A Matter of Mitochondrial Reactive Oxygen Species Generation and Citrullination and Cleavage of Histone. Front. Immunol. 8, 1849. 10.3389/fimmu.2017.01849 29375550 PMC5767187

[B173] Nathan C. (2006). Neutrophils and Immunity: Challenges and Opportunities. Nat. Rev. Immunol. 6, 173–182. 10.1038/nri1785 16498448

[B174] Németh T. Sperandio M. Mócsai A. (2020). Neutrophils as Emerging Therapeutic Targets. Nat. Rev. Drug Discov. 19, 253–275. 10.1038/s41573-019-0054-z 31969717

[B175] Nerlov C. Graf T. (1998). PU.1 Induces Myeloid Lineage Commitment in Multipotent Hematopoietic Progenitors. Genes Dev. 12, 2403–2412. 10.1101/gad.12.15.2403 9694804 PMC317050

[B176] Nordenfelt P. Tapper H. (2011). Phagosome Dynamics during Phagocytosis by Neutrophils. J. Leukoc. Biol. 90, 271–284. 10.1189/jlb.0810457 21504950

[B177] Nourshargh S. Renshaw S. A. Imhof B. A. (2016). Reverse Migration of Neutrophils: where, when, How, and Why? Trends Immunol. 37, 273–286. 10.1016/j.it.2016.03.006 27055913

[B178] Oliveira R. A. Narciso C. D. Bisinotto R. S. Perdomo M. C. Ballou M. A. Dreher M. (2010). Effects of Feeding Polyphenols from Pomegranate Extract on Health, Growth, Nutrient Digestion, and Immunocompetence of Calves. J. Dairy Sci. 93, 4280–4291. 10.3168/jds.2010-3314 20723701

[B179] Ou J. G. (1991). [Effect of Chinese Medicine "gu Chi Wan" on Chemotaxis and Phagocytosis of Polymorphonuclear Leukocytes from Peripheral Blood in Juvenile Periodontitis Patients]. Zhonghua Kou Qiang Yi Xue Za Zhi. 26, 51–53. 2032487

[B180] Panche A. N. Diwan A. D. Chandra S. R. (2016). Flavonoids: an Overview. J. Nutr. Sci. 5, e47. 10.1017/jns.2016.41 28620474 PMC5465813

[B181] Park W. H. Park S. Y. Kim H. M. Kim C. H. (2004). Effect of a Korean Traditional Formulation, Hwaotang, on Superoxide Generation in Human Neutrophils, Platelet Aggregation in Human Blood, and Nitric Oxide, Prostaglandin E2 Production and Paw Oedema Induced by Carrageenan in Mice. Immunopharmacol. Immunotoxicol. 26, 53–73. 10.1081/iph-120029945 15106732

[B182] Paula F. S. Kabeya L. M. Kanashiro A. De Figueiredo A. S. G. Azzolini A. E. C. S. Uyemura S. A. (2009). Modulation of Human Neutrophil Oxidative Metabolism and Degranulation by Extract of Tamarindus indica L. Fruit Pulp. Food Chem. Toxicol. 47, 163–170. 10.1016/j.fct.2008.10.023 19022329

[B183] Pečivová J. Mačičková T. Sviteková K. Nosáľ R. (2012). Quercetin Inhibits Degranulation and Superoxide Generation in PMA Stimulated Neutrophils. Interdiscip. Toxicol. 5, 81–83. 23118592 10.2478/v10102-012-0014-5 PMC3485658

[B184] Peng L. Li L. He X. L. Yu J. Y. Zeng Z. J. Yang W. J. (2020). Memantine Displays Antimicrobial Activity by Enhancing Escherichia coli Pathogen-Induced Formation of Neutrophil Extracellular Traps. Front. Cel Infect Microbiol. 10, 47. 10.3389/fcimb.2020.00047 PMC7031421 32117815

[B185] Perveen S. Alqahtani J. Orfali R. Aati H. Y. Al-Taweel A. M. Ibrahim T. A. (2020). Antibacterial and Antifungal Sesquiterpenoids from Aerial Parts of Anvillea Garcinii. Molecules 25. 1730. 10.3390/molecules25071730 PMC7180898 32283756

[B186] Peters A. M. Saverymuttu S. H. Keshavarzian A. Bell R. N. Lavender J. P. (1985). Splenic Pooling of Granulocytes. Clin. Sci. (Lond) 68, 283–289. 10.1042/cs0680283 3971660

[B187] Petri B. Phillipson M. Kubes P. (2008). The Physiology of Leukocyte Recruitment: an In Vivo Perspective. J. Immunol. 180, 6439–6446. 10.4049/jimmunol.180.10.6439 18453558

[B188] Phillipson M. Heit B. Colarusso P. Liu L. Ballantyne C. M. Kubes P. (2006). Intraluminal Crawling of Neutrophils to Emigration Sites: a Molecularly Distinct Process from Adhesion in the Recruitment Cascade. J. Exp. Med. 203, 2569–2575. 10.1084/jem.20060925 17116736 PMC2118150

[B189] Pillay J. Den Braber I. Vrisekoop N. Kwast L. M. De Boer R. J. Borghans J. A. M. (2010). *In vivo* labeling with 2H2O Reveals a Human Neutrophil Lifespan of 5.4 Days. Blood 116, 625–627. 10.1182/blood-2010-01-259028 20410504

[B190] Pohl K. Grimm X. A. Caceres S. M. Poch K. R. Rysavy N. Saavedra M. (2020). *Mycobacterium* Abscessus Clearance by Neutrophils Is Independent of Autophagy. Infect. Immun. 88, e00024–20. 10.1128/iai.00024-20 32423916 PMC7375761

[B191] Popov S. V. Popova G. Y. Nikolaeva S. Y. Golovchenko V. V. Ovodova R. G. (2005). Immunostimulating Activity of Pectic Polysaccharide fromBergenia Crassifolia (l.) Fritsch. Phytother. Res. 19, 1052–1056. 10.1002/ptr.1789 16372372

[B192] Ragab D. Salah Eldin H. Taeimah M. Khattab R. Salem R. (2020). The COVID-19 Cytokine Storm; what We Know So Far. Front. Immunol. 11, 1446. 10.3389/fimmu.2020.01446 32612617 PMC7308649

[B193] Ramprasath V. R. Shanthi P. Sachdanandam P. (2006). Effect ofSemecarpus Anacardium Linn. Nut Milk Extract on Rat Neutrophil Functions in Adjuvant Arthritis. Cell Biochem. Funct. 24, 333–340. 10.1002/cbf.1243 15912568

[B194] Rasheed H. M. F. Rasheed F. Qureshi A. W. Jabeen Q. (2016). Immunostimulant Activities of the Aqueous Methanolic Extract of Leptadenia Pyrotechnica , a Plant from Cholistan Desert. J. Ethnopharmacology 186, 244–250. 10.1016/j.jep.2016.03.039 26997551

[B195] Rege N. Bapat R. D. Koti R. Desai N. K. Dahanukar S. (1993). Immunotherapy with Tinospora Cordifolia: a New Lead in the Management of Obstructive Jaundice. Indian J. Gastroenterol. 12, 5–8. 8330924

[B196] Rhodes A. Evans L. E. Alhazzani W. Levy M. M. Antonelli M. Ferrer R. (2017). Surviving Sepsis Campaign: International Guidelines for Management of Sepsis and Septic Shock: 2016. Intensive Care Med. 43, 304–377. 10.1007/s00134-017-4683-6 28101605

[B197] Ríos J.-L. (2010). Effects of Triterpenes on the Immune System. J. Ethnopharmacol. 128, 1–14. 10.1016/j.jep.2009.12.045 20079412

[B198] Ross G. D. Vĕtvicka V. (1993). CR3 (CD11b, CD18): a Phagocyte and NK Cell Membrane Receptor with Multiple Ligand Specificities and Functions. Clin. Exp. Immunol. 92, 181–184. 10.1111/j.1365-2249.1993.tb03377.x 8485905 PMC1554824

[B199] Sagiv J. Y. Michaeli J. Assi S. Mishalian I. Kisos H. Levy L. (2015). Phenotypic Diversity and Plasticity in Circulating Neutrophil Subpopulations in Cancer. Cel Rep. 10, 562–573. 10.1016/j.celrep.2014.12.039 25620698

[B200] Saini A. Sharma S. Chhibber S. (2009). Induction of Resistance to Respiratory Tract Infection with Klebsiella pneumoniae in Mice Fed on a Diet Supplemented with Tulsi (Ocimum Sanctum) and Clove (Syzgium Aromaticum) Oils. J. Microbiol. Immunol. Infect. 42, 107–113. 19597641

[B201] Saitoh T. Komano J. Saitoh Y. Misawa T. Takahama M. Kozaki T. (2012). Neutrophil Extracellular Traps Mediate a Host Defense Response to Human Immunodeficiency Virus-1. Cell Host & Microbe 12, 109–116. 10.1016/j.chom.2012.05.015 22817992

[B202] Scapini P. Marini O. Tecchio C. Cassatella M. A. (2016). Human Neutrophils in the Saga of Cellular Heterogeneity: Insights and Open Questions. Immunol. Rev. 273, 48–60. 10.1111/imr.12448 27558327

[B203] Schumann J. (2016). It Is All about Fluidity: Fatty Acids and Macrophage Phagocytosis. Eur. J. Pharmacol. 785, 18–23. 10.1016/j.ejphar.2015.04.057 25987422

[B204] Segal A. W. Geisow M. Garcia R. Harper A. Miller R. (1981). The Respiratory Burst of Phagocytic Cells Is Associated with a Rise in Vacuolar pH. Nature 290, 406–409. 10.1038/290406a0 7219526

[B205] Sengeløv H. Follin P. Kjeldsen L. Lollike K. Dahlgren C. Borregaard N. (1995). Mobilization of Granules and Secretory Vesicles during In Vivo Exudation of Human Neutrophils. J. Immunol. 154, 4157–4165. 7535822

[B206] Shabeeba M. A. Rathinasamy K. (2018). Antibacterial and Anticancer Activity of the Purified Cashew Nut Shell Liquid: Implications in Cancer Chemotherapy and Wound Healing. Nat. Prod. Res. 32, 2856–2860. 28934859 10.1080/14786419.2017.1380022

[B207] Sharma V. Thakur M. Chauhan N. S. Dixit V. K. (2010). Immunomodulatory Activity of Petroleum Ether Extract ofAnacyclus Pyrethrum. Pharm. Biol. 48, 1247–1254. 10.3109/13880201003730642 20843161

[B208] Sharma U. Bala M. Kumar N. Singh B. Munshi R. K. Bhalerao S. (2012). Immunomodulatory Active Compounds from Tinospora Cordifolia. J. Ethnopharmacol. 141, 918–926. 10.1016/j.jep.2012.03.027 22472109

[B209] Shen F. Tang X. Cheng W. Wang Y. Wang C. Shi X. (2016). Fosfomycin Enhances Phagocyte-Mediated Killing of Staphylococcus aureus by Extracellular Traps and Reactive Oxygen Species. Sci. Rep. 6, 19262. 10.1038/srep19262 26778774 PMC4726045

[B210] Shi J. Gilbert G. E. Kokubo Y. Ohashi T. (2001). Role of the Liver in Regulating Numbers of Circulating Neutrophils. Blood 98, 1226–1230. 10.1182/blood.v98.4.1226 11493474

[B211] Shikov A. N. Pozharitskaya O. N. Makarova M. N. Makarov V. G. Wagner H. (2014). Bergenia Crassifolia (L.) Fritsch—Pharmacology and Phytochemistry. Phytomedicine 21, 1534–1542. 10.1016/j.phymed.2014.06.009 25442262

[B212] Shrestha S. S. Ferrarese I. Sut S. Zengin G. Grana S. Ak G. (2021). Phytochemical Investigations and *In Vitro* Bioactivity Screening on Melia Azedarach L. Leaves Extract from Nepal. Chem. Biodivers 8, 1–18. 10.1002/cbdv.202001070 33682999

[B213] Shukla S. Mehta A. John J. Mehta P. Vyas S. P. Shukla S. (2009). Immunomodulatory Activities of the Ethanolic Extract of *Caesalpinia* Bonducella Seeds. J. Ethnopharmacol. 125, 252–256. 10.1016/j.jep.2009.07.002 19607900

[B214] Sibille Y. Marchandise F. X. (1993). Pulmonary Immune Cells in Health and Disease: Polymorphonuclear Neutrophils. Eur. Respir. J. 6, 1529–1543. 8112448

[B215] Silvestre-Roig C. Hidalgo A. Soehnlein O. (2016). Neutrophil Heterogeneity: Implications for Homeostasis and Pathogenesis. Blood 127, 2173–2181. 10.1182/blood-2016-01-688887 27002116

[B216] Silvestre-Roig C. Braster Q. Wichapong K. Lee E. Y. Teulon J. M. Berrebeh N. (2019). Externalized Histone H4 Orchestrates Chronic Inflammation by Inducing Lytic Cell Death. Nature 569, 236–240. 10.1038/s41586-019-1167-6 31043745 PMC6716525

[B217] Soehnlein O. (2012). Multiple Roles for Neutrophils in Atherosclerosis. Circ. Res. 110, 875–888. 10.1161/circresaha.111.257535 22427325

[B218] Solanki Y. B. Jain S. M. (2010). Immunostimolatory Activities ofVigna mungoL. Extract in Male Sprague-Dawley Rats. J. Immunotoxicol. 7, 213–218. 10.3109/15476911003792278 20433246

[B219] Sônego F. Castanheira F. V. Ferreira R. G. Kanashiro A. Leite C. A. Nascimento D. C. (2016). Paradoxical Roles of the Neutrophil in Sepsis: Protective and Deleterious. Front. Immunol. 7, 155. 10.3389/fimmu.2016.00155 27199981 PMC4844928

[B220] Stark M. A. Huo Y. Burcin T. L. Morris M. A. Olson T. S. Ley K. (2005). Phagocytosis of Apoptotic Neutrophils Regulates Granulopoiesis via IL-23 and IL-17. Immunity 22, 285–294. 10.1016/j.immuni.2005.01.011 15780986

[B221] Stasiuk M. Kozubek A. (2010). Biological Activity of Phenolic Lipids. Cell. Mol. Life Sci. 67, 841–860. 10.1007/s00018-009-0193-1 20213924 PMC11115636

[B222] Steele C. W. Karim S. A. Leach J. D. G. Bailey P. Upstill-Goddard R. Rishi L. (2016). CXCR2 Inhibition Profoundly Suppresses Metastases and Augments Immunotherapy in Pancreatic Ductal Adenocarcinoma. Cancer Cell 29, 832–845. 10.1016/j.ccell.2016.04.014 27265504 PMC4912354

[B223] Steinhauser M. L. Kunkel S. L. Hogaboam C. M. (1999). New Frontiers in Cytokine Involvement during Experimental Sepsis. ILAR J. 40, 142–150. 10.1093/ilar.40.4.142 11406692

[B224] Sudam V. S. Potnuri A. G. Subhashini N. J. P. (2017). Syk—GTP RAC-1 Mediated Immune-Stimulatory Effect of Cuscuta Epithymum, *Ipomoea* Batata and *Euphorbia* Hirta Plant Extracts. Biomed. Pharmacother. 96, 742–749. 10.1016/j.biopha.2017.10.060 29049977

[B225] Sudha P. Asdaq S. M. Dhamingi S. S. Chandrakala G. K. (2010). Immunomodulatory Activity of Methanolic Leaf Extract of Moringa Oleifera in Animals. Indian J. Physiol. Pharmacol. 54, 133–140. 21090530

[B226] Summers C. Rankin S. M. Condliffe A. M. Singh N. Peters A. M. Chilvers E. R. (2010). Neutrophil Kinetics in Health and Disease. Trends Immunol. 31, 318–324. 10.1016/j.it.2010.05.006 20620114 PMC2930213

[B227] T Hart L. A. Nibbering P. H. Van Den Barselaar M. T. Van Dijk H. Van Den Berg A. J. Labadie R. P. (1990). Effects of Low Molecular Constituents from Aloe Vera Gel on Oxidative Metabolism and Cytotoxic and Bactericidal Activities of Human Neutrophils. Int. J. Immunopharmacol 12, 427–434. 10.1016/0192-0561(90)90026-j 2167880

[B228] Tao L. Xu M. Dai X. Ni T. Li D. Jin F. (2018). Polypharmacological Profiles Underlying the Antitumor Property of Salvia Miltiorrhiza Root (Danshen) Interfering with NOX-dependent Neutrophil Extracellular Traps. Oxid Med. Cel Longev 2018, 4908328. 10.1155/2018/4908328 PMC6120273 30210653

[B229] Templeton A. J. Mcnamara M. G. Šeruga B. Vera-Badillo F. E. Aneja P. Ocaña A. (2014). Prognostic Role of Neutrophil-To-Lymphocyte Ratio in Solid Tumors: a Systematic Review and Meta-Analysis. J. Natl. Cancer Inst. 106, dju124. 10.1093/jnci/dju124 24875653

[B230] Thatte U. M. Kulkarni M. R. Dahanukar S. A. (1992). Immunotherapeutic Modification of Escherichia coli Peritonitis and Bacteremia by Tinospora Cordifolia. J. Postgrad. Med. 38, 13–15. 1512717

[B231] Todd R. F., 3rd (1996). The Continuing Saga of Complement Receptor Type 3 (CR3). J. Clin. Invest. 98, 1–2. 10.1172/jci118752 8690779 PMC507390

[B232] Toiu A. Mocan A. Vlase L. Pârvu A. E. Vodnar D. C. Gheldiu A. M. (2019). Comparative Phytochemical Profile, Antioxidant, Antimicrobial and *In Vivo* Anti-inflammatory Activity of Different Extracts of Traditionally Used Romanian Ajuga Genevensis L. And A. Reptans L. (Lamiaceae). Molecules 24, 1597. 10.3390/molecules24081597 PMC6515068 31018502

[B233] Toshkova R. Nikolova N. Ivanova E. Ivancheva S. Serkedjieva J. (2004). *In vitro* investigation on the Effect of a Plant Preparation with Antiviral Activity on the Functions of Mice Phagocyte Cells. Pharmazie 59, 150–154. 15025186

[B234] Trevisan M. T. S. Pfundstein B. Haubner R. Würtele G. Spiegelhalder B. Bartsch H. (2006). Characterization of Alkyl Phenols in Cashew (Anacardium Occidentale) Products and Assay of Their Antioxidant Capacity. Food Chem. Toxicol. 44, 188–197. 10.1016/j.fct.2005.06.012 16095792

[B235] Urban C. F. Nett J. E. (2019). Neutrophil Extracellular Traps in Fungal Infection. Semin. Cel Dev. Biol. 89, 47–57. 10.1016/j.semcdb.2018.03.020 PMC6170733 29601861

[B236] Urban C. F. Lourido S. Zychlinsky A. (2006). How Do Microbes Evade Neutrophil Killing? Cell Microbiol. 8, 1687–1696. 10.1111/j.1462-5822.2006.00792.x 16939535

[B237] Ussov W. Y. Aktolun C. Myers M. J. Jamar F. Peters A. M. (1995). Granulocyte Margination in Bone Marrow: Comparison with Margination in the Spleen and Liver. Scand. J. Clin. Lab. Invest. 55, 87–96. 10.3109/00365519509075382 7624741

[B238] Van Der Nat J. Klerx J. Van Dijk H. De Silva K. Labadie R. (1986). Immunomodulatory Activity of an Aqueous Extract of the Stem Bark of Azadirachta indica. Planta Med. 52, 428–429. 10.1055/s-2007-969236 17345390

[B239] Wang M.-X. Liu Y. Y. Hu B. H. Wei X. H. Chang X. Sun K. (2010). Total Salvianolic Acid Improves Ischemia-Reperfusion-Induced Microcirculatory Disturbance in Rat Mesentery. Wjg 16, 5306–5316. 10.3748/wjg.v16.i42.5306 21072893 PMC2980679

[B240] Wang W. Mao S. Yu H. Wu H. Shan X. Zhang X. (2019). Pinellia Pedatisecta Lectin Exerts a Proinflammatory Activity Correlated with ROS-MAPKs/NF-Κb Pathways and the NLRP3 Inflammasome in RAW264.7 Cells Accompanied by Cell Pyroptosis. Int. Immunopharmacol. 66, 1–12. 10.1016/j.intimp.2018.11.002 30415189

[B241] Wang S. Fu L. Huang K. Han J. Zhang R. Fu Z. (2020). Neutrophil-to-lymphocyte Ratio on Admission Is an Independent Risk Factor for the Severity and Mortality in Patients with Coronavirus Disease 2019. J. Infect. 82, e16-e18. 10.1016/j.jinf.2020.09.022 32979408 PMC7513911

[B242] Wang J. Ma Y. Guo M. Yang H. Guan X. (2021). Salvianolic Acid B Suppresses EMT and Apoptosis to Lessen Drug Resistance through AKT/mTOR in Gastric Cancer Cells. Cytotechnology 73, 49–61. 10.1007/s10616-020-00441-4 33505113 PMC7817758

[B243] Wickramasinghe R. Kumara R. R. De Silva E. D. Ratnasooriya W. D. Handunnetti S. (2014). Inhibition of Phagocytic and Intracellular Killing Activity of Human Neutrophils by Aqueous and Methanolic Leaf Extracts of Ixora Coccinea. J. Ethnopharmacol. 153, 900–907. 10.1016/j.jep.2014.03.064 24704593

[B244] Xu L. Zhang W. Kwak M. Zhang L. Lee P. C. W. Jin J. O. (2019). Protective Effect of Melatonin against Polymicrobial Sepsis Is Mediated by the Anti-bacterial Effect of Neutrophils. Front. Immunol. 10, 1371. 10.3389/fimmu.2019.01371 31275316 PMC6593141

[B245] Yamaryo T. Oishi K. Yoshimine H. Tsuchihashi Y. Matsushima K. Nagatake T. (2003). Fourteen-member Macrolides Promote the Phosphatidylserine Receptor-dependent Phagocytosis of Apoptotic Neutrophils by Alveolar Macrophages. Aac 47, 48–53. 10.1128/aac.47.1.48-53.2003 PMC148990 12499168

[B246] Yang S.-H. Hong C.-Y. Yu C.-L. (2002). The Stimulatory Effects of Nasal Discharge from Patients with Perennial Allergic Rhinitis on Normal Human Neutrophils Are Normalized after Treatment with a New Mixed Formula of Chinese Herbs. Int. Immunopharmacol. 2, 1627–1639. 10.1016/s1567-5769(02)00133-9 12469937

[B247] Yaseen R. Branitzki-Heinemann K. Moubasher H. Setzer W. N. Naim H. Y. Von Köckritz-Blickwede M. (2017). *In Vitro* Testing of Crude Natural Plant Extracts from Costa Rica for Their Ability to Boost Innate Immune Cells against Staphylococcus aureus. Biomedicines 5. 10.3390/biomedicines5030040 PMC5618298 28678207

[B248] Yasuda H. Sonoda A. Yamamoto M. Kawashima Y. Takishita Y. Morita A. (2019). 17-β-estradiol Enhances Neutrophil Extracellular Trap Formation by Interaction with Estrogen Membrane Receptor. Arch. Biochem. Biophys. 663, 64–70. 10.1016/j.abb.2018.12.028 30590021

[B249] Yates C. R. Bruno E. J. Yates M. E. D. (2021). Tinospora Cordifolia: A Review of its Immunomodulatory Properties. J. Diet. Suppl., 1–15. 10.1080/19390211.2021.1873214 33480818

[B250] Yipp B. G. Kubes P. (2013). NETosis: How Vital Is it? Blood 122, 2784–2794. 10.1182/blood-2013-04-457671 24009232

[B251] Yipp B. G. Kim J. H. Lima R. Zbytnuik L. D. Petri B. Swanlund N. (2017). The Lung Is a Host Defense Niche for Immediate Neutrophil-Mediated Vascular Protection. Sci. Immunol. 2, eaam8929. 10.1126/sciimmunol.aam8929 28626833 PMC5472445

[B252] Yu Y. Koehn C. D. Yue Y. Li S. Thiele G. M. Hearth-Holmes M. P. (2015). Celastrol Inhibits Inflammatory Stimuli-Induced Neutrophil Extracellular Trap Formation. Cmm 15, 401–410. 10.2174/1566524015666150505160743 PMC4527119 25941817

[B253] Zaporozhets T. S. Besednova N. N. Liamkin G. P. Loenko Iu N. Popov A. M. (1991). [Immunomodulating Properties of Pectin from Seawater Grass Zostera]. Antibiot. Khimioter 36, 31–34. 1755708

[B254] Zhang D. Chen G. Manwani D. Mortha A. Xu C. Faith J. J. (2015). Neutrophil Ageing Is Regulated by the Microbiome. Nature 525, 528–532. 10.1038/nature15367 26374999 PMC4712631

[B255] Zhang H. Qiu S. L. Tang Q. Y. Zhou X. Zhang J. Q. He Z. Y. (2019). Erythromycin Suppresses Neutrophil Extracellular Traps in Smoking-Related Chronic Pulmonary Inflammation. Cell Death Dis. 10, 678. 10.1038/s41419-019-1909-2 31515489 PMC6742640

[B256] Zhang R. Ji Y. Zhang X. Kennelly E. J. Long C. (2020a). Ethnopharmacology of *Hypericum* Species in China: A Comprehensive Review on Ethnobotany, Phytochemistry and Pharmacology. J. Ethnopharmacol. 254, 112686. 10.1016/j.jep.2020.112686 32101776

[B257] Zhang S. Li L. Shen A. Chen Y. Qi Z. (2020b). Rational Use of Tocilizumab in the Treatment of Novel Coronavirus Pneumonia. Clin. Drug Investig. 40, 511–518. 10.1007/s40261-020-00917-3 PMC7183818 32337664

